# Computation-guided scaffold exploration of 2*E*,6*E*-1,10-*trans/cis-*eunicellanes

**DOI:** 10.3762/bjoc.20.115

**Published:** 2024-06-07

**Authors:** Zining Li, Sana Jindani, Volga Kojasoy, Teresa Ortega, Erin M Marshall, Khalil A Abboud, Sandra Loesgen, Dean J Tantillo, Jeffrey D Rudolf

**Affiliations:** 1 Department of Chemistry, University of Florida, PO Box 117200, Gainesville, FL 32611, USA; 2 Department of Chemistry, University of California–Davis, 1 Shields Ave., Davis, CA 95616, USA; 3 Academy of Scientific and Innovative Research (AcSIR), Ghaziabad, 201002, India; 4 Whitney Laboratory for Marine Bioscience, University of Florida, 9505 N Ocean Shore Blvd., St. Augustine, FL 32080, USA

**Keywords:** atropisomer, Cope rearrangement, DFT calculations, diterpene, electrophilic cyclization, eunicellane

## Abstract

Eunicellane diterpenoids are a unique family of natural products containing a foundational 6/10-bicyclic framework and can be divided into two main classes, *cis* and *trans*, based on the configurations of their ring fusion at C1 and C10. Previous studies on two bacterial diterpene synthases, Bnd4 and AlbS, revealed that these enzymes form *cis*- and *trans*-eunicellane skeletons, respectively. Although the structures of these diterpenes only differed in their configuration at a single position, C1, they displayed distinct chemical and thermal reactivities. Here, we used a combination of quantum chemical calculations and chemical transformations to probe their intrinsic properties, which result in protonation-initiated cyclization, Cope rearrangement, and atropisomerism. Finally, we exploited the reactivity of the *trans*-eunicellane skeleton to generate a series of 6/6/6 gersemiane-type diterpenes via electrophilic cyclization.

## Introduction

**Figure 1:**
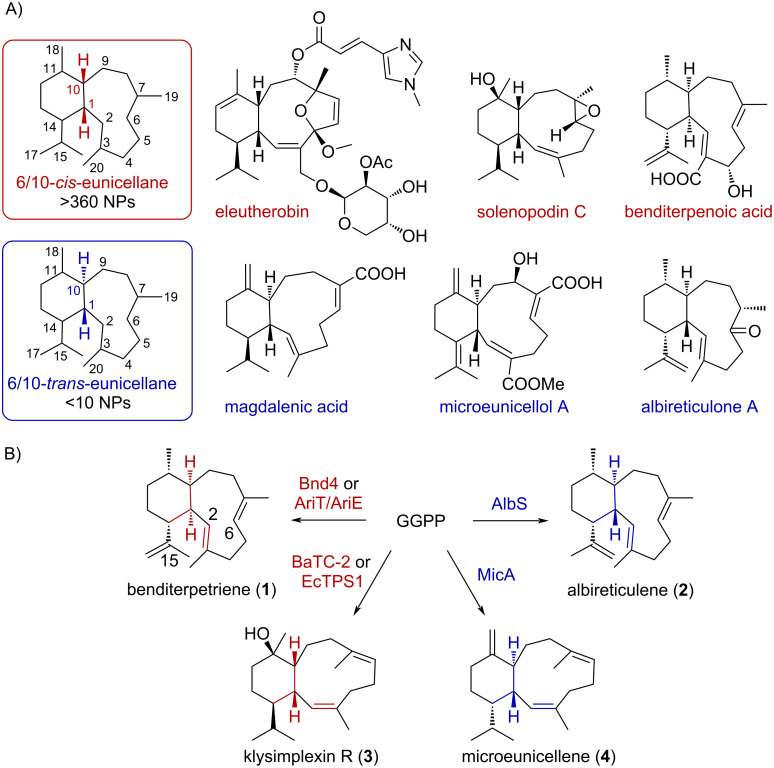
Eunicellane diterpenoids and their biosyntheses. (A) The 6/10-bicyclic hydrocarbon framework is conserved in eunicellane diterpenoids. Selected natural products consisting of *cis-* (red) and *trans-*eunicellane skeletons (blue) are shown. (B) Four types of diterpene synthases are known to form the eunicellane skeleton, each with differing configurations at the ring-fused carbons and the 2,3-alkene.

The eunicellane diterpenoids are a family of nearly 400 natural products that present a conserved 6/10-bicyclic hydrocarbon framework [1–3]. Mostly known from soft corals [2], but with a growing number of family members in bacteria and plants [4], these diterpenoids have four main structural differences: the number and location of oxidized carbons, the absence or presence of transannular ether bridges, the configuration (*cis* or *trans*) of the bicyclic ring fusion, and the presence and configuration (*E* or *Z*) of alkenes in the 10-membered ring (Figure 1A). During biosynthesis, the eunicellane skeleton is first constructed by terpene synthases that cyclize the diterpene precursor geranylgeranyl diphosphate (Figure 1B) [5–9]. The latter two structural elements, the configurations of the 6/10 system and the C2–C3 alkene, are instilled by these terpene synthases.

Four types of eunicellane synthases are known (Figure 1B). The first eunicellane synthase identified, Bnd4 from the biosynthesis of benditerpenoic acid in *Streptomyces* sp. (CL12-4) [5], forms a *cis*-eunicellane named benditerpetriene (**1**) [6]. In **1**, the C2–C3 and C6–C7 alkenes are *E*-configured, with the latter alkene configuration being conserved in all known eunicellane cyclization mechanisms. The first *trans*-eunicellane synthase, AlbS from the biosynthesis of albireticulone in *Streptomyces albireticuli* [10], was also identified from bacteria and forms albireticulene (**2**), a C1 diastereomer of **1** that also features the 2*E* alkene [7]. Two coral enzymes, BaTC-2 and EcTPS1, were found to form klysimplexin R (**3**), a 2*Z*-*cis*-eunicellane [8–9]. Recently, a third bacterial version, MicA, was identified as producing the 2*Z*-*trans*-eunicellane (**4**, microeunicellene) necessary for microeunicellol biosynthesis in *Micromonospora* sp. HM134 [11].

During our work on Bnd4, AlbS, and their enzymatic products **1** and **2**, we noticed that the *cis* and *trans*-eunicellane skeletons behaved differently under certain conditions. For example, we successfully collected NMR data of **1** in chloroform [5], but when we dissolved **2** in chloroform for NMR, it cyclized into two 6/6/6-tricyclic diterpenes (**5** and **6**) [7]. We discovered that **2** was much more sensitive to acid than **1** and eventually took advantage of its reactivity to determine its absolute configuration [7]. Here, we sought to understand the molecular basis of chemical and thermal reactivities of these diterpene skeletons. We also took advantage of their intrinsic chemical properties to transform the eunicellanes in functionalized tricyclic skeletons of natural product importance.

## Results and Discussion

### Ring fusion configuration does not affect protonation-induced cyclization

**Figure 2:**
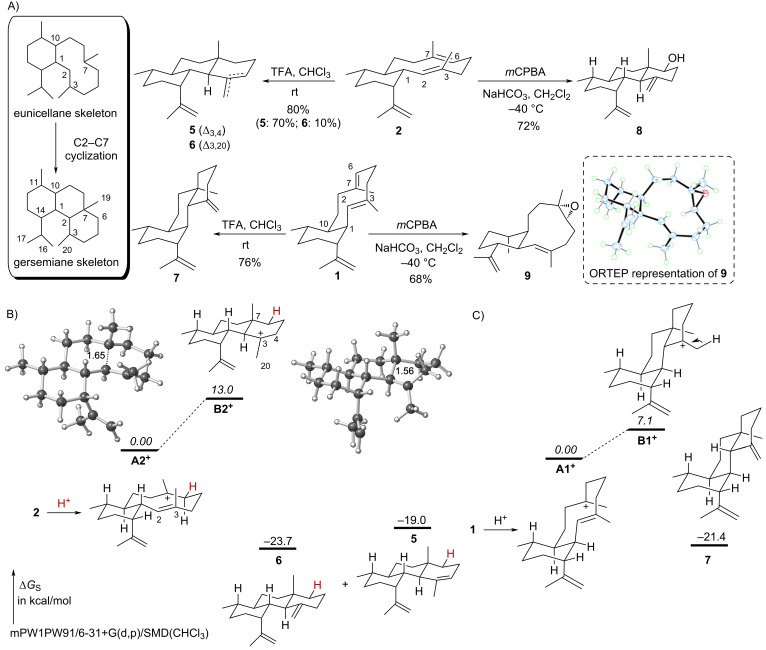
Protonation-mediated cyclization of *trans*- and *cis*-eunicellanes. (A) The 2*E*-*trans-* and 2*E*-*cis*-eunicellane skeletons form 6/6/6-tricyclic gersemiene skeletons in the presence of acid. Epoxidation of **1** with *m*CPBA yields the epoxide **9**, but the similar reaction with **2** yields gersemienol **8.** Isolation yields are provided. (B and C) Results of DFT calculations on the protonation-induced cyclizations of **1** and **2**. The energies of the cationic intermediates (italicized values) are not on the same energy scale as for the substrates and products (i.e., the free energy of **B2^+^** is relative to **A2^+^**, while those of **5** and **6** are relative to that of **2**; the free energy of **B1^+^** is relative to **A1^+^**, while that of **7** is relative to **1**; the energies given in B and C are not relative to each other). Note that the other rotamer of **7** is predicted to be 3.5 kcal mol^−1^ higher in energy than the one shown, an energy difference that is larger than expected based on experiment.

Our initial observation that albireticulene (**2**) was unstable in chloroform, resulting in two tricyclic isomers gersemienes A (**5**) and B (**6**) [7], while benditerpetriene (**1**) was stable [5], led us to investigate the protonation-induced cyclization of the eunicellane skeleton (Figure 2). When **1** and **2** were individually subjected to acidic conditions (i.e., TFA in CHCl_3_), both cyclized into 6/6/6-tricyclic skeletons via a selective C2–C7 cyclization after protonation at C6 to give *trans*-BC ring systems (i.e., *cis*,*trans*-6/6/6 for **1** and *trans*,*trans*-6/6/6 for **2**; Figure 2A). Only a single isomer, with the exocyclic olefin on ring C, of *cis*,*trans*-gersemiene was found, which matched a previous report of **1** cyclization in aqueous 0.1 M HCl into gersemiene C (**7**, Figures S1–S3, Supporting Information File 1) [12].

To assess these chemically-induced cationic cyclization mechanisms, we performed quantum chemical calculations [mPW1PW91/6–31+G(d/p)/SMD(chloroform)] [13–20] to obtain the relative free energies of the cationic intermediates and transition states that interconvert them, as well as the relative free energies of the neutral products. In the cyclization of **2**, protonation at C6, results in a C7 tertiary cationic intermediate (**A2^+^**) where C2 is only 1.65 Å away from C7 (Figure 2B); this structure can be viewed as protonated **5** or **6** with a strongly hyperconjugated C2–C7 bond [21]. Reducing this hyperconjugation via bending the exocyclic methyl group such that the formal *p*-orbital at C3 no longer aligns with the C2–C7 bond results in the 6/6/6-tricyclic C3 tertiary cation **B2^+^**, which is 13.0 kcal mol^−1^ higher in energy. In principle, either **A2^+^** or **B2^+^** could be deprotonated to form products. A similar energy profile is seen for protonation-induced cyclization of **1**, although the 6/6/6-tricyclic C3 tertiary cation is 7.1 kcal mol^−1^ higher in energy than its preceding intermediate (Figure 2C). A lower energy conformer of **1** exists, but we have been unable to find the corresponding cation **A1b^+^**, which implies that protonation and cyclization may be concerted for that conformer (Figure S4, Supporting Information File 1).

We previously transformed **2** into the *trans*,*trans*-6/6/6-tricyclic C6 alcohol **8** using *m*CPBA to epoxidize the C6–C7 alkene [7]. Under the same conditions, epoxidation of **1** yielded the 6,7-epoxide **9**, which readily crystallized (Figure 2A and Figures S5–S10, Table S1 in Supporting Information File 1). Similarly, the 6,7-epoxy derivatives of klysimplexin R (**3**) and microeunicellene (**4**) were recently synthesized and isolated [11,21]; **3** cyclized to the 6/6/6-scaffold after the addition of acid [22]. An X-ray structure of **9** allowed us to solve its absolute configuration and measure the C2–C7 distance to be 3.31 Å. This distance matches reasonably well with the calculated distance, 3.36 Å, of the lowest energy conformer of **9**. Calculation of the proposed 6,7-epoxy derivative of **2**, which was never isolated, gave a similar estimated C2–C7 distance of 3.57 Å, suggesting that a factor other than distance controls any subsequent or concomitant cyclization.

### Cope rearrangement is facile for *trans*-eunicellanes

**Figure 3:**
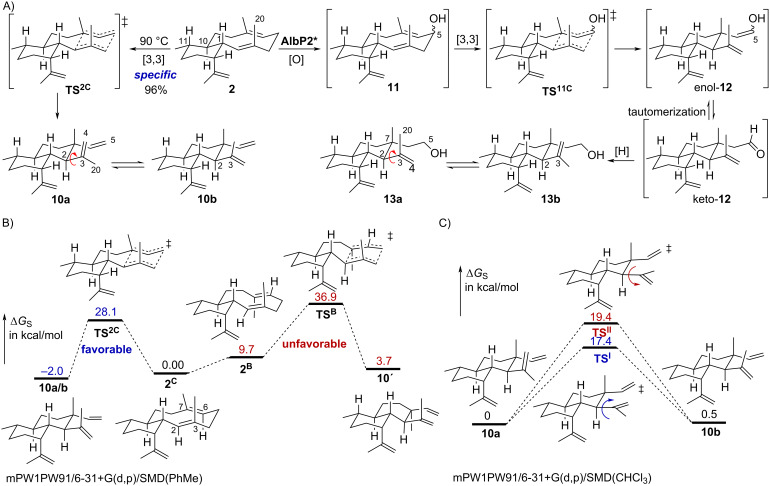
Cope rearrangement in *trans*-eunicellanes. (A) The 2*E*-*trans-*eunicellane undergoes thermal Cope rearrangement, yielding atropisomers **10a**/**10b**; an isolation yield of 96% was previously reported [10]. A biosynthetically related analog is similarly proposed to undergo oxy-Cope rearrangement to yield **13a**/**13b**. (B) Results of DFT calculations indicate that the Cope rearrangement of **2** is more favorable via a chair–chair transition state than a chair–boat transition state. (C) Results of DFT calculations on the atropisomers **10a**/**10b** and the free energy barriers for clockwise (blue) and counter-clockwise (red) rotations.

During our study of the albireticulene (**2**) biosynthetic gene cluster, we found that **2** easily undergoes Cope rearrangement at 90 °C to generate the stereospecific 6/6-bicyclic product **10** in 96% yield (Figure 3A) [10]. This Cope rearrangement product was found as two inseparable atropisomers (**10a**/**10b**) at room temperature, which coalesced into a single conformer at 130 °C [10]. We anticipated that **1** would similarly undergo Cope rearrangement. However, when we heated **1** up to 200 °C for 5 h, we did not observe any Cope rearrangement products and we were able to recover >90% of **1**. DFT calculations [mPW1PW91/6–31+G(d/p)/SMD(toluene)] on the Cope rearrangement of **2** revealed a free energy barrier through the chair–chair transition state of 28.1 kcal mol^−1^ and an overall Δ*G* from **2** to **10a**/**10b**, which have the same predicted energy, of −2.0 kcal mol^−1^ (Figure 3B). The relative free energy barrier through the chair–boat transition state was calculated to be 36.9 kcal mol^−1^, but would require a prior chair-to-boat conformational change of 9.7 kcal mol^−1^, providing a similar activation barrier for the Cope rearrangement step itself (27.2 kcal mol^−1^). For **1**, a potential Cope rearrangement was predicted to be 32.5 kcal mol^−1^, an overall higher energetic barrier compared to **2** (Figure S11, Supporting Information File 1). The lowest transition state for a Cope rearrangement, a chair–chair structure at 29.3 kcal mol^−1^, originates from a DD (down–down orientations of the methyl groups on the 10-membered ring) conformer that is 3.2 kcal mol^−1^ higher in free energy than the most dominant conformer, leading to the predicted overall barrier of 32.5 kcal mol^−1^ and consistent with the diminished reactivity observed experimentally. The two lowest energy conformers of **1** (−3.2 and −3.0 kcal mol^−1^ relative to the DD conformer mostly likely to undergo Cope rearrangement) would both require significantly higher free energy barriers of 38.0 and 38.8 kcal mol^−1^, respectively, to react through their associated chair–boat Cope transition states (Figure S11, Supporting Information File 1). In addition, the resulting products were predicted to be approximately 10 kcal mol^−1^ higher in free energies than the reactants. We note here, circumstantially, that no Cope rearrangement products of *cis*-eunicellanes have been reported. Overall, the *trans* ring fusion of the 2*E*,6*E-trans-*eunicellanes appears to control whether Cope rearrangement is energetically possible.

In the same biosynthetic study [10], we also identified what appeared to be an oxy-Cope product (**13a**/**13b**), which would logically originate from a 5-hydroxyalbireticulene analog (**11**, Figure 3A). Because this product was isolated directly from the producing bacterium as inseparable atropisomers (i.e., no heat was applied), we speculated that a lower activation energy may allow for oxy-Cope rearrangement at 28 °C. DFT calculations (in toluene) of 5-hydroxyalbireticulene for both the pseudo-axial and pseudo-equatorial conformations of the C5 hydroxy moiety suggest that there is not a significant difference in the free energy barriers for the oxy-Cope rearrangement (27.7 kcal mol^−1^; Figure S12, Supporting Information File 1) of uncharged **11** compared to that of **2** (Figure 3B). The free energy barrier value for **11** is likely too high to be non-enzymatic at 28 °C and may at least require deprotonation of the hydroxy group or at least H-bonding with solvent [23].

In regard to the atropisomerism of **10a**/**10b**, the two atropisomers are approximately equivalent in energy (0.5 kcal mol^−1^ difference) and require at least 17.4 kcal mol^−1^ to undergo conversion (Figure 3C). Interestingly, rotation in different directions requires barriers that differ by 2 kcal mol^−1^.

### Scaffold exploration of the eunicellane skeleton

**Figure 4:**
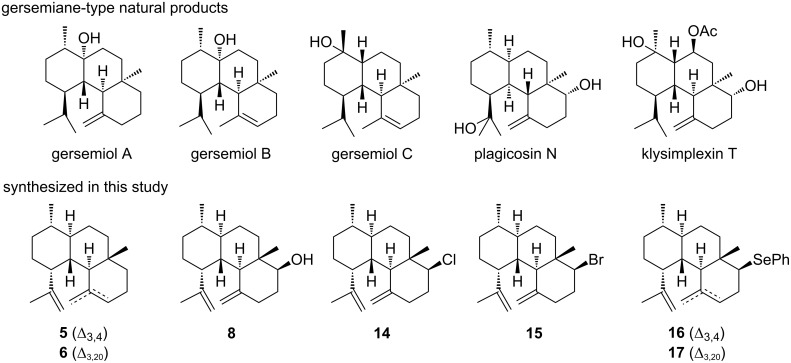
Scaffold exploration of the 2*E*-*trans-*eunicellane skeleton.

During protonation-induced cyclization of **1** and **2**, the C6–C7 alkene showed higher nucleophilicity than either of the other two double bonds likely due to the unique conformation of the eunicellane skeleton. This selective reactivity was further supported when the C6–C7 alkene of **2** was oxidized by *m*CPBA oxidation, resulting in formation of the gersemianol derivative **8** [7]. Inspired by the reactivity of the *trans*-eunicellane skeleton, we conducted a series of chemical transformations to convert **2** into *trans*/*trans*-6/6/6 bicyclic skeletons with various functional groups at C6 (Figure 4). The goals were to evaluate the scope of electrophile-mediated cyclization and diversify the gersemiane skeleton for bioactivity assays. A structural similarity search of **5** and **6** revealed that several natural products, including the coral gersemiols [24] and plagicosin N from liverwort [25], possess a similar 6/6/6-skeleton and likely originate from a *cis*-eunicellane skeleton.

We first independently tested **2** with halogen electrophiles NCS and NBS (1.2 equivalents). NCS and NBS provided the C6-monohalogenated 6/6/6-tricyclic products **14** and **15** in 78% and 74% yields (Figure 4 and Figures S13–S23 in Supporting Information File 1). The ^1^H NMR chemical shifts for H6 of 6-chlorogersemiene (**14**), 3.81 ppm (*J* = 12.1, 4.8 Hz), and 6-bromogersemiene (**15**), 4.03 ppm (*J* = 12.4, 4.7 Hz), supported their assignments. The large coupling constants (>10 Hz) of H6 support its axial position and therefore the equatorial nature of the halogen. These configurations were also verified by NOESY correlations between H6 and H2 in both compounds. Some minor isomers of the halogenated derivatives were detected by LC–MS, but were deemed too low in abundance to purify. Two gersemiane derivatives (**16** and **17**), in a ratio of 3:1 in 82% overall yield, with a phenylselenyl groups at C6 were obtained when **2** was reacted with PhSeBr in acetonitrile overnight (Figure 4 and Figures S24–S36 in Supporting Information File 1). A similar reaction with PhSCl was also tested but yielded only several minor products and mostly starting material, hence we decided these products were not worth further purification. The minor isomers of the halogenated derivatives were presumably detected but in too low yields to purify. Since 6/6/6-tricyclic diterpenes are known to show cytotoxicity [26], we tested our seven analogs for cytotoxicity against human colon carcinoma HCT-116 cells. Unfortunately, none of the compounds up to 10 μM showed any activity when tested in MTT-based cell viability assays.

## Conclusion

Eunicellane diterpenoids have been known for over 50 years, but it was not until recently that their biosynthetic precursors, the eunicellane skeletons, were identified and isolated. Currently, four different eunicellane skeletons are known: 2*E*-*cis* (**1**), 2*E*- *trans* (**2**), 2*Z*-*cis* (**3**), and 2*Z*-*trans* (**4**). Based on the current distribution of eunicellane diterpenoids isolated, the coral-derived 2*Z*-*cis*-eunicellane appears to be the dominant form in nature (≈98%). Perhaps the inherent reactivity seen for 2*E*-*trans*-eunicellanes contributes to their presumed rarity in nature, as certain conditions or transformations, either enzymatic or spontaneous, may alter the hydrocarbon skeleton. This idea is supported by the recent discovery of the aridacins, which transforms a 2*E*-*cis*-eunicellane into a 6/7/5-tricyclic diterpene via a cytochrome P450 [12]. Coincidentally, these reactive skeletons also provide chemists the ability to access synthetically challenging scaffolds with simple electrophilic cyclizations. With genome mining of bacterial eunicellane synthases suggesting there are a significant number of both *cis*- and *trans*-eunicellane diterpenoids remaining to be discovered [5,10–12], we expect continuing synthetic and biosynthetic advancements in eunicellane diterpenoids in the near future.

## Supporting Information

File 1 Experimental methods, NMR and MS spectra, and crystallographic information.

## Data Availability

All data that supports the findings of this study is available in the published article and/or the supporting information to this article. A data set collection of computational results is available in the ioChem-BD repository and can be accessed via https://doi.org/10.19061/iochem-bd-6-334. Crystal data for compound 9 was deposited into the CCDC, deposition number 2326275.
