# Supplementary material for: Computation-guided scaffold exploration of 2E,6E-1,10-trans/cis-eunicellanes
Source: Beilstein J Org Chem. 2024 Jun 7;20:1320–6. doi: 10.3762/bjoc.20.115 (PMC11181210; doi:10.3762/bjoc.20.115)
Supplement: File 1 — Experimental methods, NMR and MS spectra, and crystallographic information. [file Beilstein_J_Org_Chem-20-1320-s001.pdf]

BEILSTEIN JOURNAL OF ORGANIC CHEMISTRY

Supporting Information

for

Computation-guided scaffold exploration of 2*E*,6*E*-1,10-*trans*/*cis*-eunicellanes

Zining Li, Sana Jindani, Volga Kojasoy, Teresa Ortega, Erin M. Marshall, Khalil A. Abboud, Sandra Loesgen, Dean J. Tantillo and Jeffrey D. Rudolf

Beilstein J. Org. Chem. **2024,** *20,* 1320–1326. doi:10.3762/bjoc.20.115

Experimental methods, NMR and MS spectra, and crystallographic information

License and Terms: This is a supporting information file under the terms of the Creative Commons Attribution License (https://creativecommons.org/licenses/by/4.0). Please note that the reuse, redistribution and reproduction in particular requires that the author(s) and source are credited and that individual graphics may be subject to special legal provisions.
The license is subject to the *Beilstein Journal of Organic Chemistry* terms and conditions: (https://www.beilstein-journals.org/bjoc/terms)

Table of Contents

Methods S2

Figures S1–S2. ^{1}H NMR (600 MHz) and ^{13}C NMR (151 MHz) spectroscopic data for **7** S6
Figure S3. EIMS of **7** S8
Figure S4. Alternative protonation-mediated cyclization of **1** S9
Figures S5–S10. 1D and 2D NMR spectra of **9** in chloroform-*d* S9
Figure S11. Relative free energy profiles for the Cope rearrangement in *cis*-eunicellanes S17
Figure S12. Relative free energy profiles for the oxy-Cope rearrangement in **11** S18
Figures S13–S17. 1D and 2D NMR spectra of **14** in chloroform-*d* S19
Figures S18–S23. 1D and 2D NMR spectra of **15** in chloroform-*d* S23
Figures S24–S29. 1D and 2D NMR spectra of **16** in chloroform-*d* S29
Figures S30–S36. 1D and 2D NMR spectra of **17** in chloroform-*d* S35

Table S1. Crystal data and structure refinement for **9** S42

Supporting References S43

General experimental procedures. All ^{1}H, ^{13}C, 1D selective TOCSY, 1D NOE, and 2D NMR (^{1}H-^{1}H COSY, ^{1}H-^{13}C HSQC, ^{1}H-^{13}C HMBC, TOCSY and NOESY) experiments were run on a Bruker Avance III HD (600 MHz for ^{1}H and 150 MHz for ^{13}C nuclei). All NMR chemical shifts were referenced to residual solvent peaks or to $Si(CH_3)_4$ as an internal standard: spectra recorded in $CDCl_3$ were referenced to residual $CHCl_3$ at 7.26 ppm for ^{1}H or 77.00 ppm for ^{13}C; spectra recorded in C_6D_6 were referenced to residual C_6D_5H at 7.16 ppm for ^{1}H or 128.06 ppm for ^{13}C. Chemical reactions were monitored by thin layer chromatography (TLC) and high-performance liquid chromatography (HPLC). TLC was performed with 0.25 mm silica gel plates (60 F_{254}) using short-wave UV light to visualize, and I_2 or $KMnO_4$ and heat as developing agents. HPLC was performed on an Agilent 1260 Infinity LC equipped with an Agilent Zorbax SB-C18 column (150 mm × 4.6 mm, 5 µm). Preparative HPLC was carried out on an Agilent 1260 Infinity LC equipped with an Agilent Eclipse XDB-C18 column (250 mm × 21.2 mm, 7 µm). GC–MS analysis was carried out using Thermo Scientific Trace GC ultra-ISQ spectrometer with a DB-5MS glass capillary column (Agilent Technologies, 15 m × 0.25 mm i.d. and 1 µm film). High-resolution MS were recorded with Agilent 6546 QTOF mass spectrometer with an electrospray ionization (ESI) source (Agilent Technologies). Optical rotations were measured using a JASCO P-2000 polarimeter. Melting points were measured using a DigiMelt Melting Point Apparatus (MPA) 160 (Stanford Research Systems).

Synthesis of 1, 2, 5, 6, and 8

The detailed procedures to produce **1, 2, 5, 6**, and **8** were operated according to previously reported literature [1,2].

Synthesis of gersemiene C (7)

To a solution of **1** (27 mg, 0.10 mmol) in CH_3Cl (5 mL), trifluoroacetic acid (TFA, 10 µL, 0.13 mmol) was added at 0 °C and the mixture was stirred at 0 °C for 30 min, then warmed up to room temperature. A saturated Na_2CO_3 aqueous solution was added to quench the reaction after **1** was completely consumed as determined by TLC. The reaction mixture was concentrated in vacuo. The concentrated extract was purified by silica gel chromatography with a gradient elution of hexane/EtOAc (100:0, 95:5) to give the single isomer product **7** as a colorless oil (20.6 mg, 76%); R_f = 0.8 (hexanes); $[\alpha]_D^{21}$ = +64 (c = 0.10, $CHCl_3$); ^{1}H NMR (600 MHz, $CDCl_3$) δ 4.91 (d, J = 1.6 Hz, 1 H), 4.86 (d, J = 12.7 Hz, 2 H), 4.32 (s, 1 H), 2.69 (d, J = 5.0 Hz, 1 H), 2.31 (d, J = 12.0 Hz, 1 H), 2.28 (m, 1 H), 2.16 (m, 1 H), 2.00-1.92 (m, 1 H), 1.84-1.78 (m, 1 H), 1.74 (s, 3 H), 1.7-1.6 (m, 4 H), 1.57-1.47 (m, 3 H), 1.44-1.37 (m, 3 H), 1.36-1.30 (m, 1 H), 1.15 (dt, J = 13.1, 3.4 Hz, 1 H), 1.12-1.04(m, 1 H), 0.82 (s, 3 H), 0.82 (d, J = 6.5 Hz, 3 H); ^{13}C NMR (151 MHz, $CDCl_3$) δ 148.77, 148.04, 110.15, 106.86, 48.95, 42.71, 39.56, 39.24, 39.03, 37.74, 35.38, 33.93, 31.16, 28.41, 25.03, 23.83, 23.23, 22.84, 20.37, 17.94. GC–MS analysis see Fig. S3. All spectroscopic data matches the literature [3,4].

Synthesis of 9

To a solution of **1** (27 mg, 0.10 mmol) and $NaHCO_3$ (30 mg, 0.36 mmol) in CH_2Cl_2 (10 mL), *m*CPBA (75% w/w, 28 mg, 0.16 mmol) was added at −40 °C and the mixture was stirred for 30 min. A saturated Na_2SO_3 aqueous solution was added to quench the reaction after **1** was completely consumed as determined by TLC. The reaction mixture was extracted with ethyl

acetate and the combined organic extract was concentrated in vacuo. The concentrated extract was purified by silica gel chromatography with a gradient elution of hexane/EtOAc (100:0, 95:5, 80:20) to give the major product **9** as a colorless oil (19.4 mg, 68%); R_f = 0.68 (hexanes:EtOAc = 4:1); $[\alpha]_D^{21}$ = +51 (c = 0.10, $CHCl_3$); ^{1}H NMR (600 MHz, $CDCl_3$) δ 5.04 (dd, *J* = 9.8, 1.6 Hz, 1 H), 4.68 (m, 1 H), 4.66 (m, 1 H), 2.62 (dd, *J* = 9.3, 5.9 Hz, 1 H), 2.52 (ddd, *J* = 11.4, 9.7, 4.1 Hz, 1 H), 2.35-2.26 (m, 1 H), 2.21-2.12 (m, 3 H), 2.00 (td, *J* = 11.9, 3.7 Hz, 1 H), 1.70-1.64 (m, 2 H), 1.66 (d, *J* = 1.5 Hz, 3 H), 1.64-1.62 (m, 1 H), 1.61 (s, 3 H), 1.55-1.47 (m, 1 H), 1.44-1.38 (m, 1 H), 1.37-1.32 (m, 2 H), 1.30 (s, 3 H), 1.28-1.22 (m, 2 H), 1.08 (d, *J* = 7.1 Hz, 3 H), 0.91 (t, 1 H); ^{13}C NMR (151 MHz, CDCl3) δ 149.28, 132.21, 131.35, 110.24, 61.87, 60.19, 47.93, 46.21, 42.15, 35.75, 34.99, 34.88, 26.89, 26.24, 25.93, 25.84, 18.91, 18.73, 16.69, 15.64. HRESIMS: calcd for $C_{20}H_{33}O$ $[M + H]^+$ *m/z* 289.2526; found *m/z* 289.2523.

Synthesis of 6-chlorogersemiene (14):

To a solution of **2** (27 mg, 0.10 mmol) in CH_2Cl_2 (10 mL), NCS (16 mg, 0.12 mmol) was added at −40 °C and the mixture was stirred overnight and warmed up to room temperature. A saturated Na_2SO_3 aqueous solution was added to quench the reaction after **2** was detected to be completely consumed by HPLC. The reaction mixture was extracted with ethyl acetate and the combined organic extract was concentrated under reduced pressure. The concentrated extract was purified by silica gel chromatography with a gradient elution of hexane/EtOAc (100:0, 95:5) to give the major product **14** (23.5 mg, 78%) as a light yellow oil.

6-Chlorogersemiene (14): R_f = 0.88 (hexanes); $[\alpha]_D^{21}$ = +8.9 (c = 0.01, $CHCl_3$); ^{1}H NMR (600 MHz, $CDCl_3$) δ 4.96 (s, 1 H), 4.83 (s, 1 H), 4.76 (s, 1 H), 4.52 (s, 1 H), 3.81 (dd, J = 12.1, 4.8 Hz, 1 H), 2.89 (m, 1 H), 2.27-2.22 (m, 1 H), 2.14-2.07 (m, 1 H), 2.04-2.0 (m, 1 H), 1.92-1.84 (m, 2 H), 1.83-1.78 (m, 2 H), 1.71 (s, 3 H), 1.70-1.67 (m, 1 H), 1.63-1.54 (m, 2 H), 1.51-1.45 (m, 1 H), 1.40-1.33 (m, 1 H), 1.27-1.22 (m, 1 H), 1.19-1.14 (m, 1 H), 1.07-1.02 (m, 1 H), 1.00-0.92(m, 1 H), 0.92 (d, *J* = 6.4 Hz, 3 H), 0.80 (s, 3 H); ^{13}C NMR (151 MHz, $CDCl_3$) δ 147.86, 145.71, 113.25, 108.79, 72.54, 51.31, 42.53, 41.76, 40.89, 39.76, 38.12, 38.10, 37.72, 35.22, 31.15, 30.41, 26.52, 25.15, 20.08, 12.93;

Synthesis of 6-bromogersemiene (15)

To a solution of **2** (27 mg, 0.10 mmol) in CH_2Cl_2 (10 mL), NBS (21.2 mg, 0.12 mmol) was added at −40 °C and the mixture was stirred overnight and then warmed up to room temperature. A saturated Na_2SO_3 aqueous solution was added to quench the reaction after **2** was detected to be completely consumed by HPLC. The reaction mixture was extracted with ethyl acetate and the combined organic extract was concentrated under reduced pressure. The concentrated extract was purified by silica gel chromatography with a gradient elution of hexane/EtOAc (100:0, 95:5) to give the major product **15** (25.7 mg, 74%) as a light yellow oil.

6-Bromogersemiene (15): R_f = 0.9 (hexanes); $[\alpha]_D^{21}$ = +13 (c = 0.01, $CHCl_3$); ^{1}H NMR (600 MHz, $CDCl_3$) δ 4.96 (s, 1 H), 4.83 (s, 1 H), 4.76 (s, 1 H), 4.52 (s, 1 H), 4.03 (dd, *J* = 12.4, 4.7 Hz, 1 H), 2.89 (s, 1 H), 2.28-2.20 (m, 2 H), 2.14-2.03 (m, 1 H), 2.01-1.92 (m, 2 H), 1.82-1.76 (m, 1 H), 1.71 (s, 3 H), 1.71-1.67 (m, 1 H), 1.63-1.54 (m, 2 H), 1.51-1.44 (m, 1 H), 1.40-1.31 (m, 1 H), 1.29-1.11 (m, 2 H), 1.09-1.00 (m, 1 H), 1.00-0.92(m, 2 H), 0.92 (d, *J* = 6.4 Hz, 3 H), 0.84 (s,

3 H); ^{13}C NMR (151 MHz, $CDCl_3$) δ 147.82, 145.58, 113.27, 108.84, 68.38, 51.64, 42.65, 41.80, 41.27, 39.75, 39.57, 39.02, 38.09, 36.34, 31.15, 30.39, 26.50, 25.28, 20.06, 13.99.

Synthesis of 6-phenylselenylgersemienes 16 and 17

To a solution of **2** (27 mg, 0.10 mmol) in acetonitrile (20 mL), PhSeBr (26 mg, 0.11 mmol) was added at −40 °C and the mixture was stirred overnight and then warmed up to room temperature. A saturated Na_2SO_3 aqueous solution was added to quench the reaction after **2** was detected to be completely consumed by HPLC. The reaction mixture was extracted with ethyl acetate and the combined organic extract was concentrated under reduced pressure. The concentrated extract was purified by silica gel chromatography with a gradient elution of hexane/EtOAc (100:0, 95:5) to give two light yellow oils in a ratio of 3:1: the major product was **17** (26.1 mg, 61%), the minor product was **16** (9.1 mg, 21%).

6-Phenylselenylgersemiene A (16): R_f = 0.85 (hexanes); $[\alpha]_D^{21}$ = +11 (c = 0.02, $CHCl_3$); ^{1}H NMR (600 MHz, $CDCl_3$) δ 7.57 (m, 2 H), 7.24 (m, 3 H), 5.34 (s, 1 H), 4.94 (s, 1 H), 4.88 (s, 1 H), 3.38 (t, *J* = 8.6 Hz, 1 H), 2.86 (s, 1 H), 2.60-2.52 (m, 1 H), 2.43-2.34 (m, 1 H), 2.27 (d, *J* = 11.9 Hz, 1 H), 2.13 (dd, *J* = 10.1, 4.0 Hz, 1 H), 2.07-1.97 (m, 1 H), 1.85 (s, 3 H), 1.81-1.75 (m, 1 H), 1.75 (s, 3 H), 1.64-1.53 (m, 2 H), 1.50-1.44 (m, 1 H), 1.34-1.24 (m, 2 H), 1.22-1.15 (m, 1 H), 1.04-0.98 (m, 2 H), 0.90 (d, *J* = 6.4 Hz, 3 H), 0.87 (s, 3 H); ^{13}C NMR (151 MHz, $CDCl_3$) δ 147.07, 136.87, 134.59, 130.63, 128.83, 126.98, 124.78, 114.06, 56.75, 47.77, 43.17, 42.78, 42.38, 39.75, 39.48, 38.75, 34.35, 31.12, 30.73, 26.66, 25.10, 23.87, 20.37, 14.82; HRESIMS: calcd for $C_{26}H_{37}Se$ $[M - H]^-$ *m*/*z* 427.1909; found *m*/*z* 427.1907.

6-Phenylselenylgersemiene B (17): R_f = 0.88 (hexanes); $[\alpha]_D^{21}$ = +37 (c = 0.05, $CHCl_3$); ^{1}H NMR (600 MHz, $CDCl_3$) δ 7.60 (m, 2 H), 7.28 (m, 3 H), 4.94 (s, 1 H), 4.83 (s, 1 H), 4.78 (s, 1 H), 4.49 (s, 1 H), 3.10 (dd, *J* = 12.3, 4.5 Hz, 1 H), 2.92 (m, 1 H), 2.28-2.22 (m, 2 H), 2.14-2.11 (m, 1 H), 2.01-1.90 (m, 3 H), 1.84-1.81 (m, 1 H), 1.73 (s, 3 H), 1.71 (m, 1 H), 1.64-1.55 (m, 2 H), 1.52-1.47 (m, 1 H), 1.43-1.34 (m, 1 H), 1.30-1.23 (m, 2 H), 1.20-1.11 (m, 2 H), 1.04-0.98 (m, 1 H), 0.94 (d, *J* = 6.4 Hz, 3 H), 0.85 (s, 3 H); ^{13}C NMR (151 MHz, $CDCl_3$) δ 148.07, 146.90, 134.73, 130.64, 128.85, 127.07, 113.13, 107.73, 60.54, 53.01, 42.65, 41.56, 41.09, 40.23, 39.67, 39.64, 38.13, 34.80, 31.21, 30.47, 26.58, 25.53, 20.09, 15.11; HRESIMS: calcd for $C_{26}H_{37}Se$ $[M - H]^-$ *m*/*z* 427.1909; found *m*/*z* 427.1910.

X-ray diffraction structure of 9

For crystallization of **9**, pure **9** was dissolved in a 500 μL mixture of DCM:THF:CH_3OH (4:3:3) at a concentration of 20 g mL^{-1}. After two weeks of solvent evaporation at 4 °C, colorless crystals appeared. Mp = 117.3 – 119.0 °C.

X-ray experimental: X-ray Intensity data were collected at 100 K on a Bruker Dual micro source D8 Venture diffractometer and PHOTON III detector running APEX3 software package of programs and using CuKα radiation (λ = 1.54178 Å). The data frames were integrated and multi-scan scaling was applied in APEX3. Intrinsic phasing structure solution provided all of the non-H atoms.

The structure was refined using full-matrix least-squares refinement (SHELXL) [5]. The non-H atoms were refined with anisotropic displacement parameters and all of the H atoms were calculated in idealized positions and refined riding on their parent atoms. The asymmetric unit is the full hydrocarbon molecule with an epoxide, O1, bound at C6 and C7. The absolute configuration was determined with anomalous dispersion and refined with a Flack parameter of -0.01. In the final cycle of refinement, 3029 reflections (of which 2992 are observed with $I > 2\sigma(I)$) were used to refine 194 parameters and the resulting R_1, wR_2 and S (goodness of fit) were 2.68%, 6.73% and 1.062, respectively. The refinement was carried out by minimizing the wR_2 function using F^2 rather than F values. R_1 is calculated to provide a reference to the conventional R value but its function is not minimized.

Assessment of cell toxicity

Compounds **5**, **6**, **8**, and **14–17** were tested for cell viability inhibition against human colorectal carcinoma (HCT-116, ATCC CCL-247) following established protocols [6]. Cells were maintained in MEM growth media, supplemented with 10% (v/v) fetal bovine serum, penicillin (100 U mL^{-1}) and streptomycin (100 μg mL^{-1}), in a humidified chamber at 37 °C in 5% CO_2. Cells were plated into 96-well plates (7,000 cells $well^{-1}$) and maintained overnight before treatment. Cell viability was determined by measuring the reduction of the tetrazolium salt MTT [3-(4, 5-dimethylthiazolyl-2)-2,5-diphenyltetrazolium bromide] by metabolically active cells. Compounds were prepared at 10 μM in DMSO for single dose treatment. After 48 h, MTT reagent (5 mg mL^{-1} in PBS) was added to each well at a final concentration of 0.5 mg mL^{-1}. The plates were incubated for 2 h at 37°C. The growth media was removed, and then purple formazan product solubilized by the addition of 50 μL DMSO. Absorbance was measured at 550 nm using a Biotek Synergy 96-well plate reader. Metabolic activity of vehicle-treated cells (0.1% DMSO) was defined as 100% cell growth. Etoposide (250 μM) was used as a positive control.

Figure S1. ^{1}H NMR spectrum of **7** in chloroform-*d* (600 MHz).

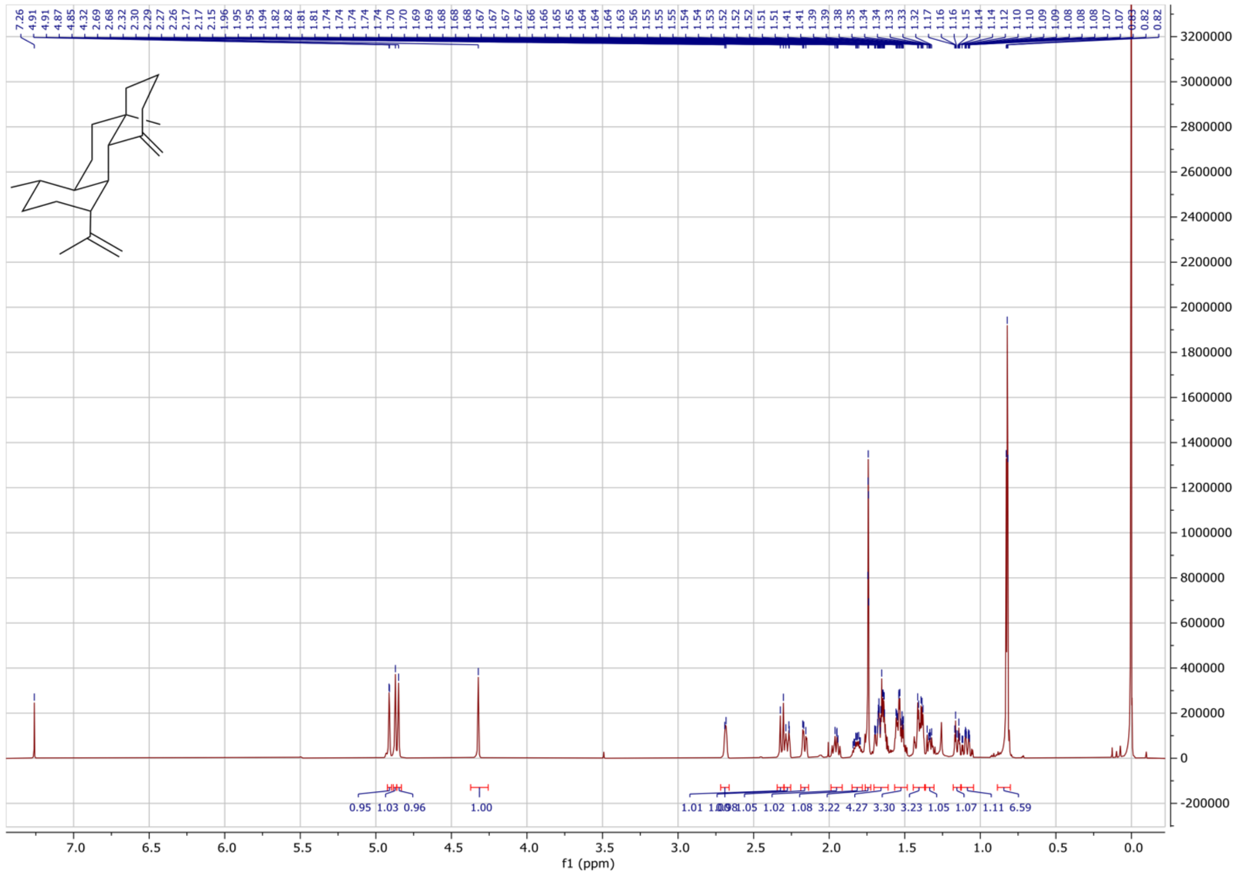

Figure S2. ^{13}C NMR spectrum of **7** in chloroform-*d* (151 MHz).

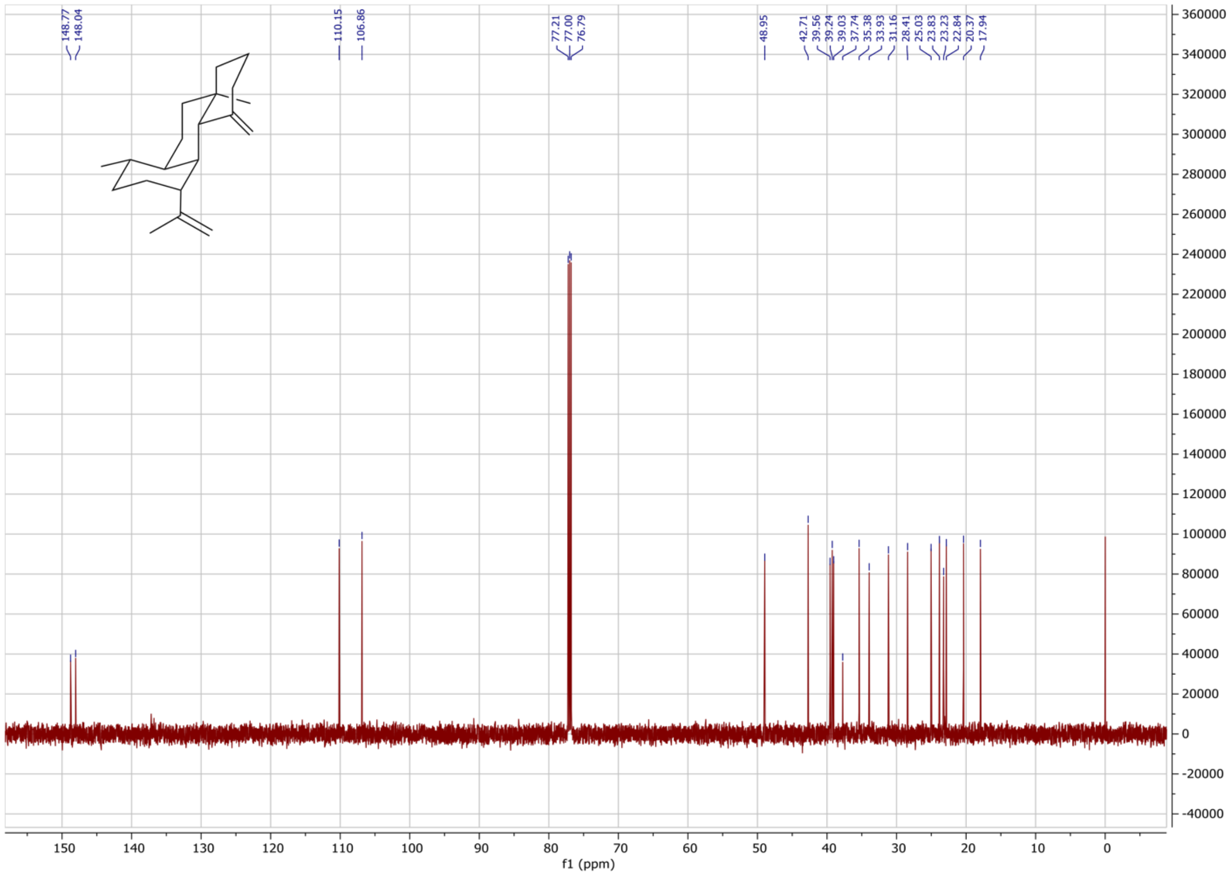

Figure S3. EIMS of gersemiene C (**7**). The fragmentation matched the recently reported spectrum [3].

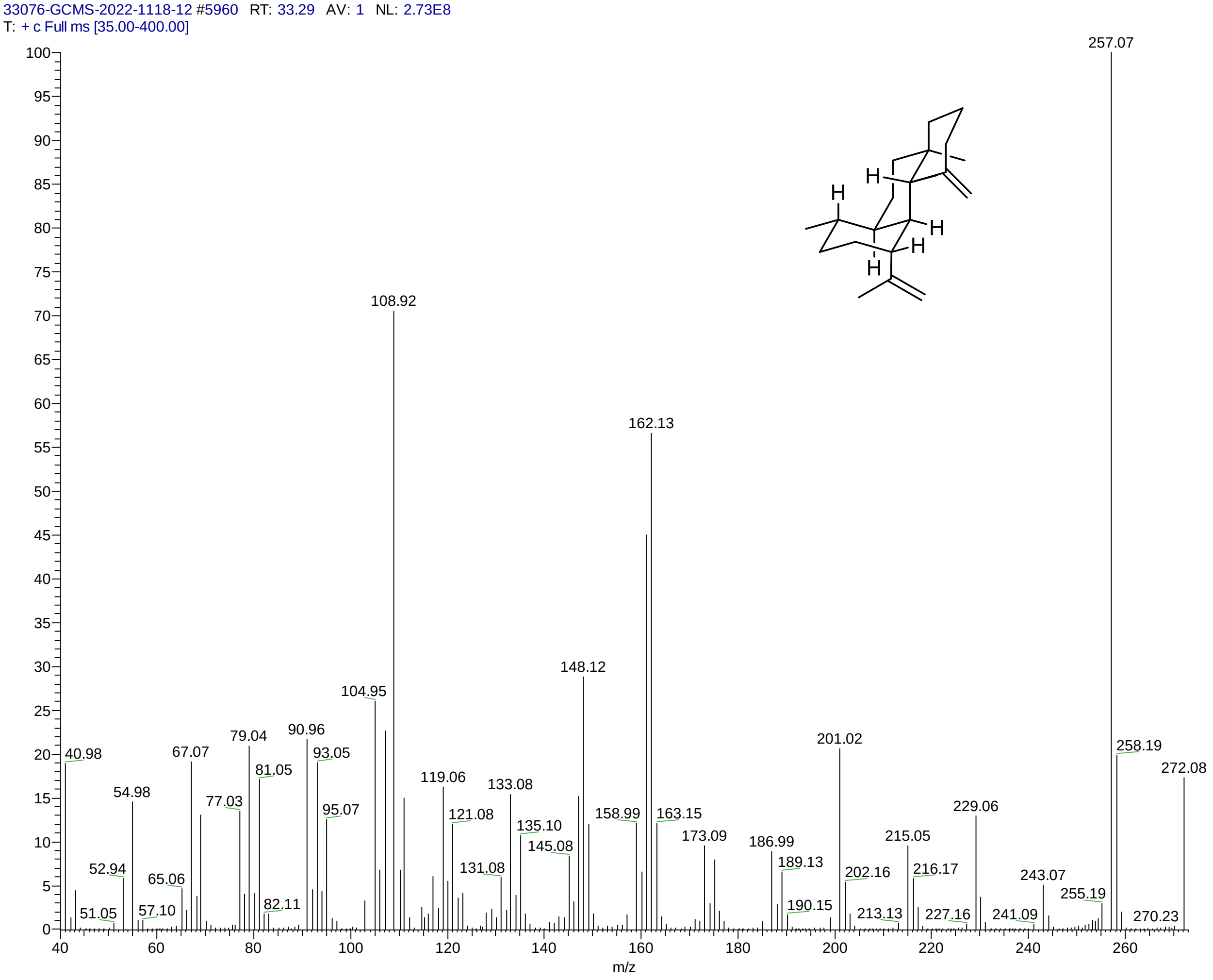

Figure S4. Alternative protonation-mediated cyclization of **1**. Results of DFT calculations on the protonation-induced cyclizations of **1**. The free energies (blue values) are compared with the values of similar structures in Figure 2C. The free energies of B1b$^+$ and B1$^+$ are relative to A1b$^+$ and A1$^+$, respectively, while those of **7** and the unnumbered putative product are relative to that of **1**. A similar scheme to that found in Fig. 2C is included here for comparison.

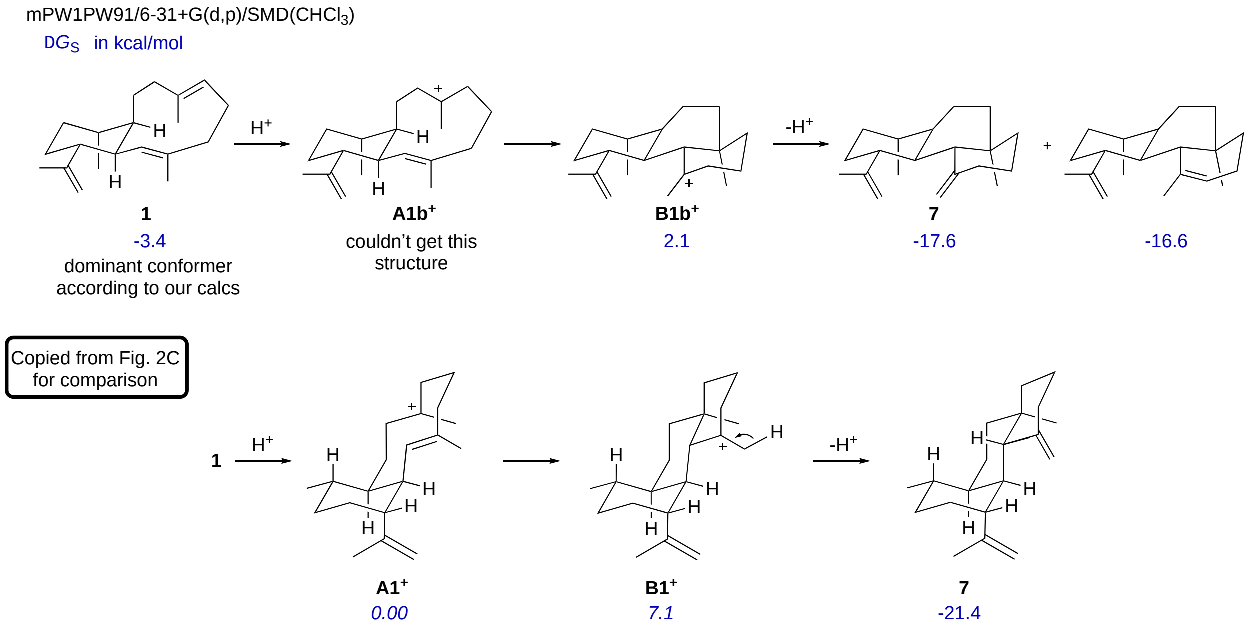

Figure S5. ^{1}H NMR spectrum of **9** in chloroform-*d* (600 MHz).

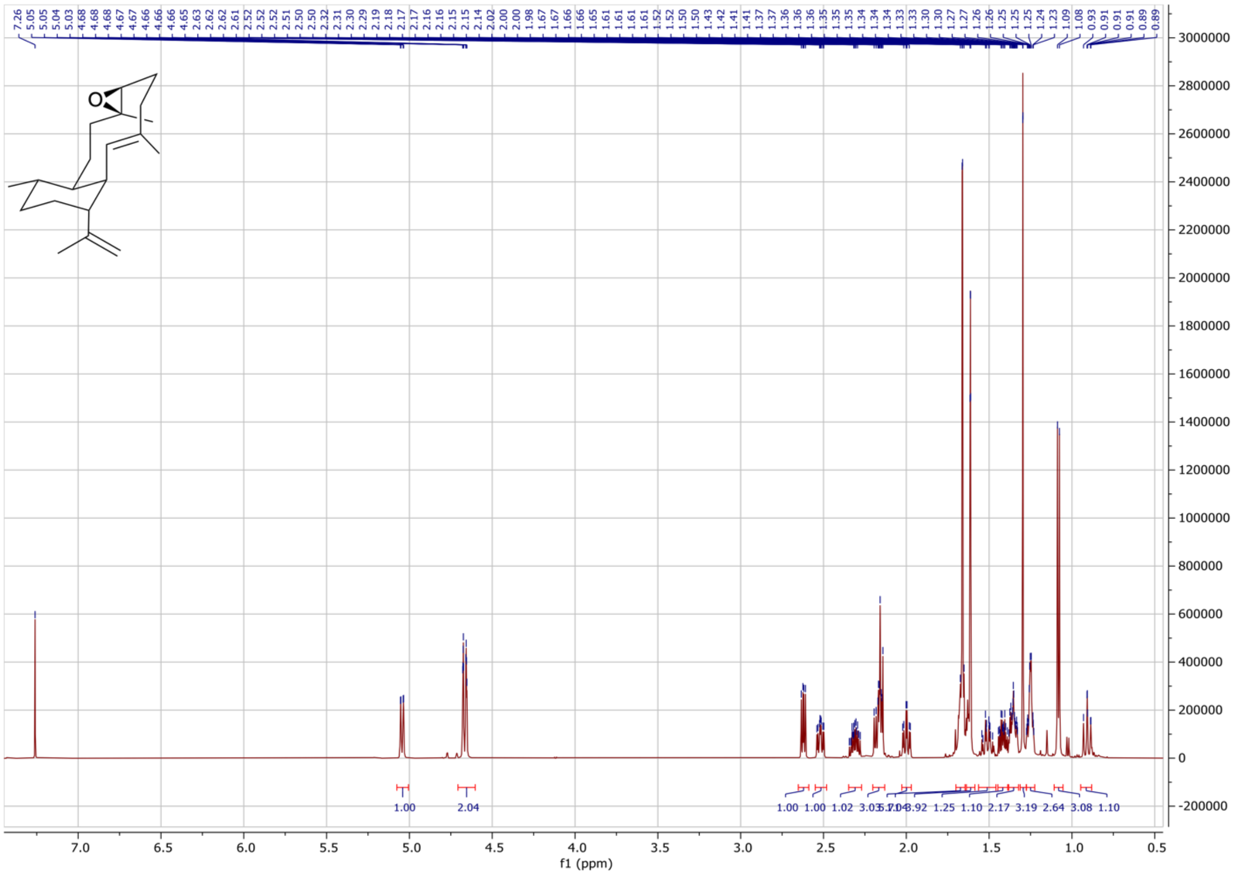

Figure S6. ^{13}C NMR spectrum of **9** in chloroform-*d* (151 MHz).

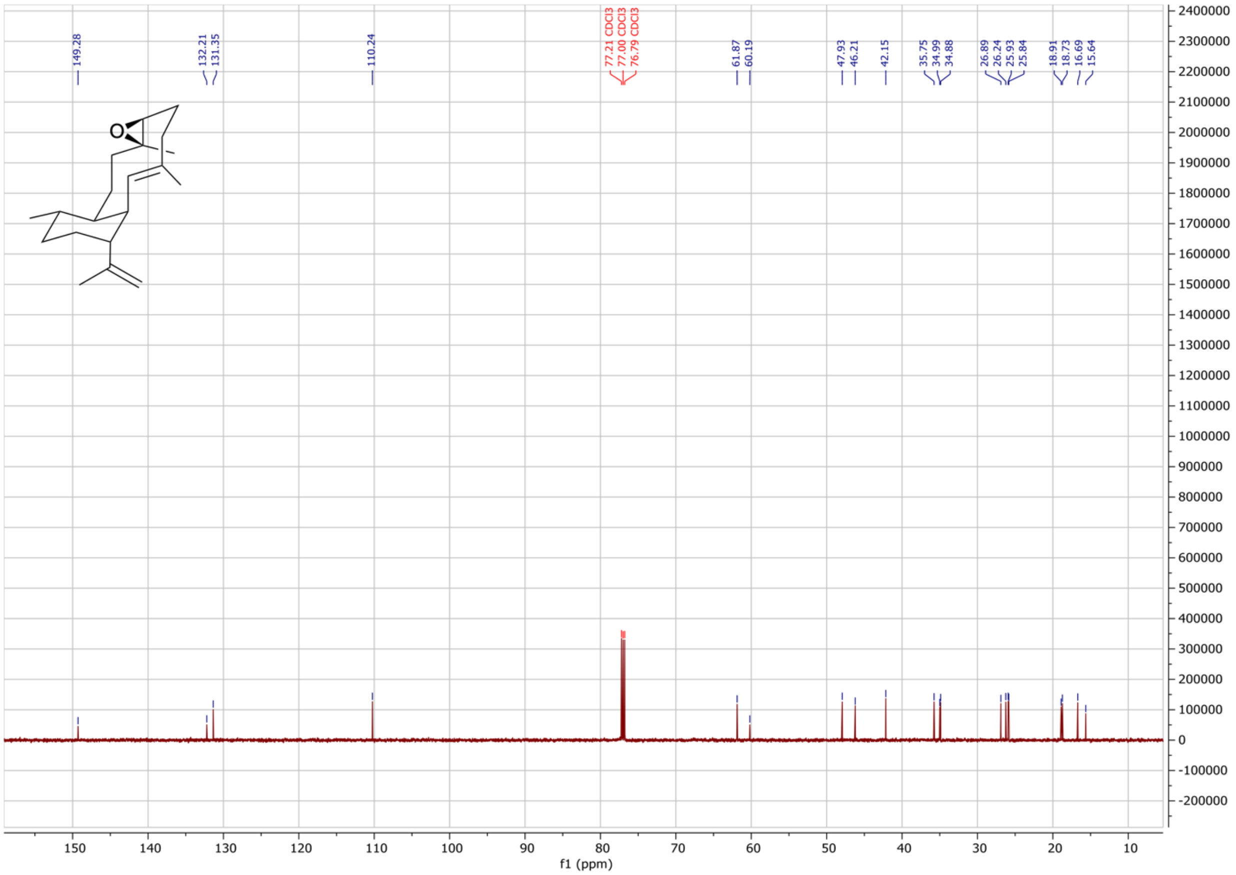

Figure S7. ¹H-¹H COSY spectrum of **9** in chloroform-*d* (600 MHz).

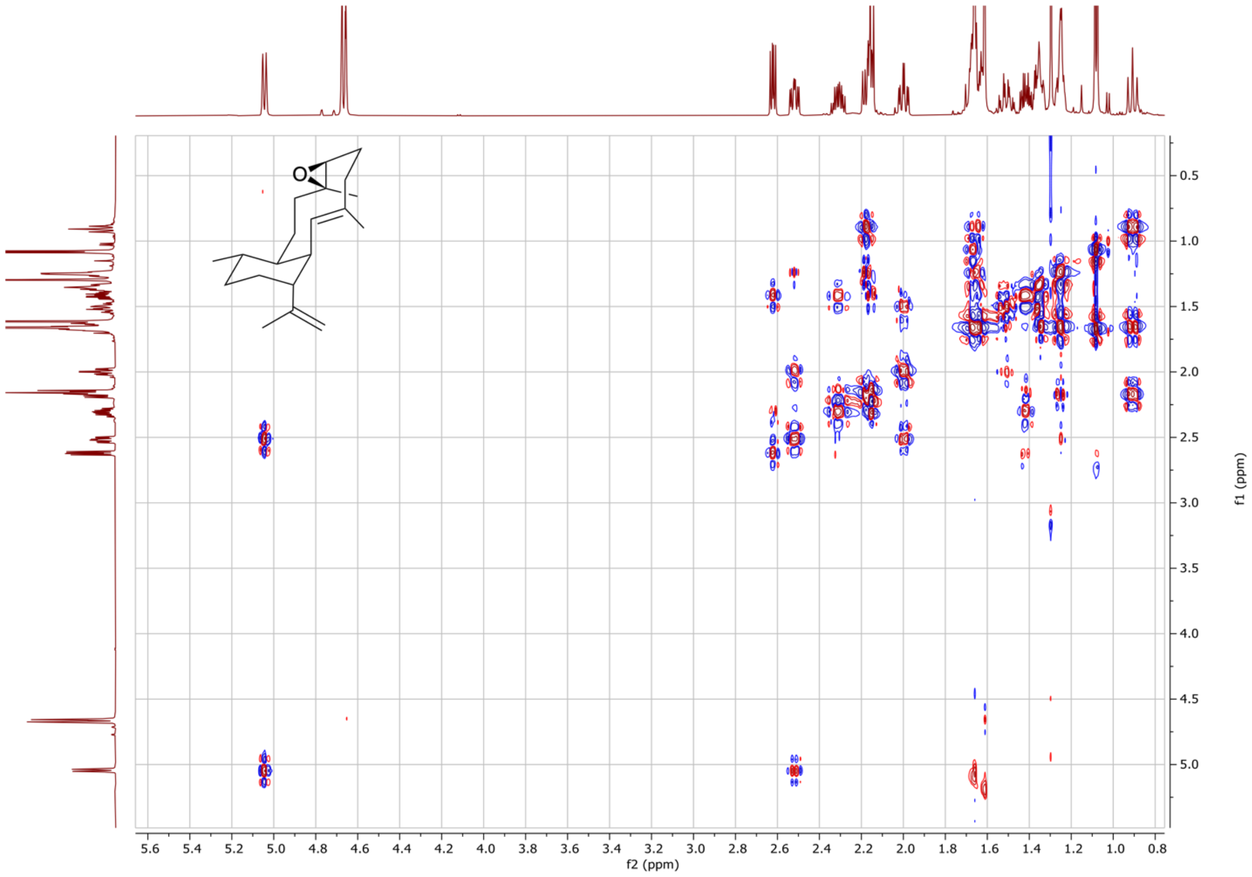

Figure S8. ^{1}H-^{13}C HSQC spectrum of **9** in chloroform-*d* (600 MHz).

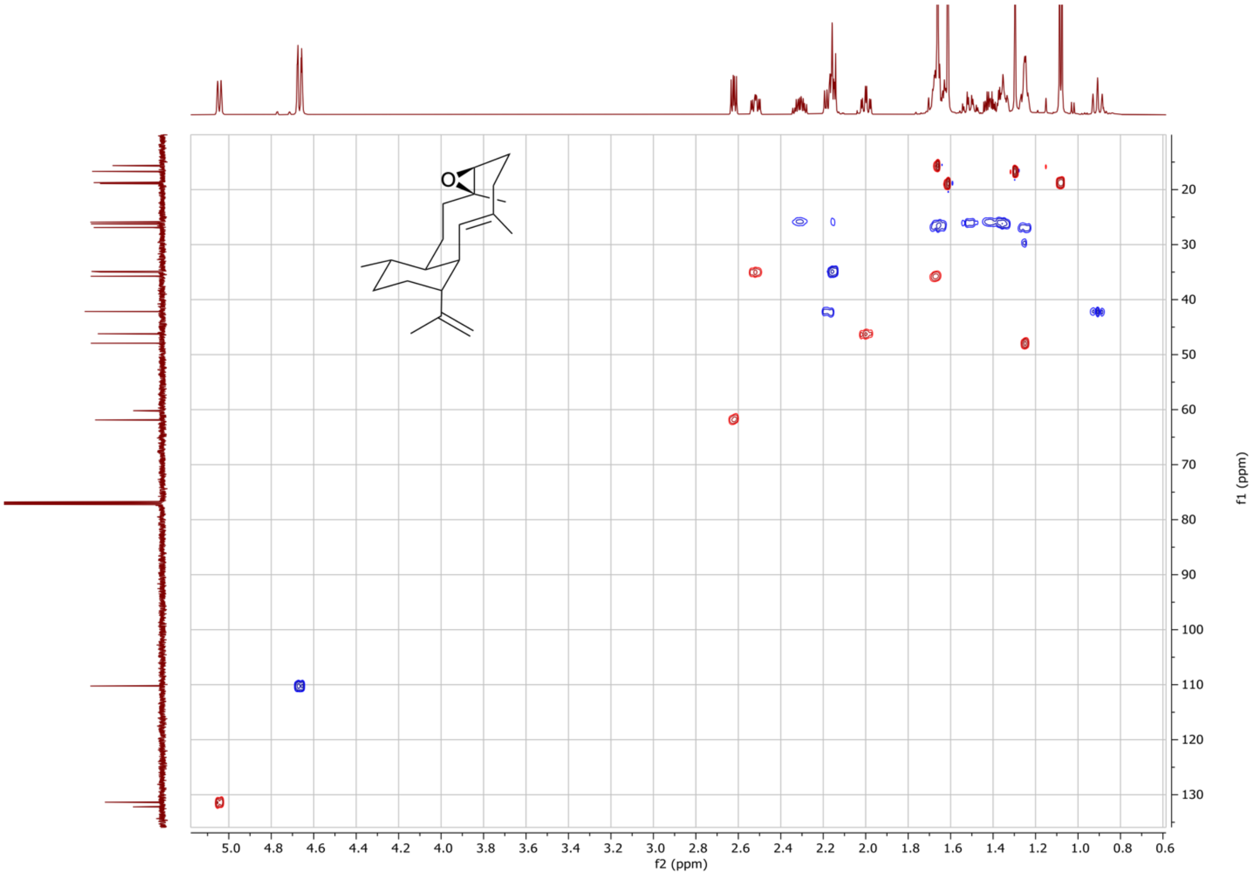

Figure S9. ^{1}H-^{13}C HMBC spectrum of **9** in chloroform-*d* (600 MHz).

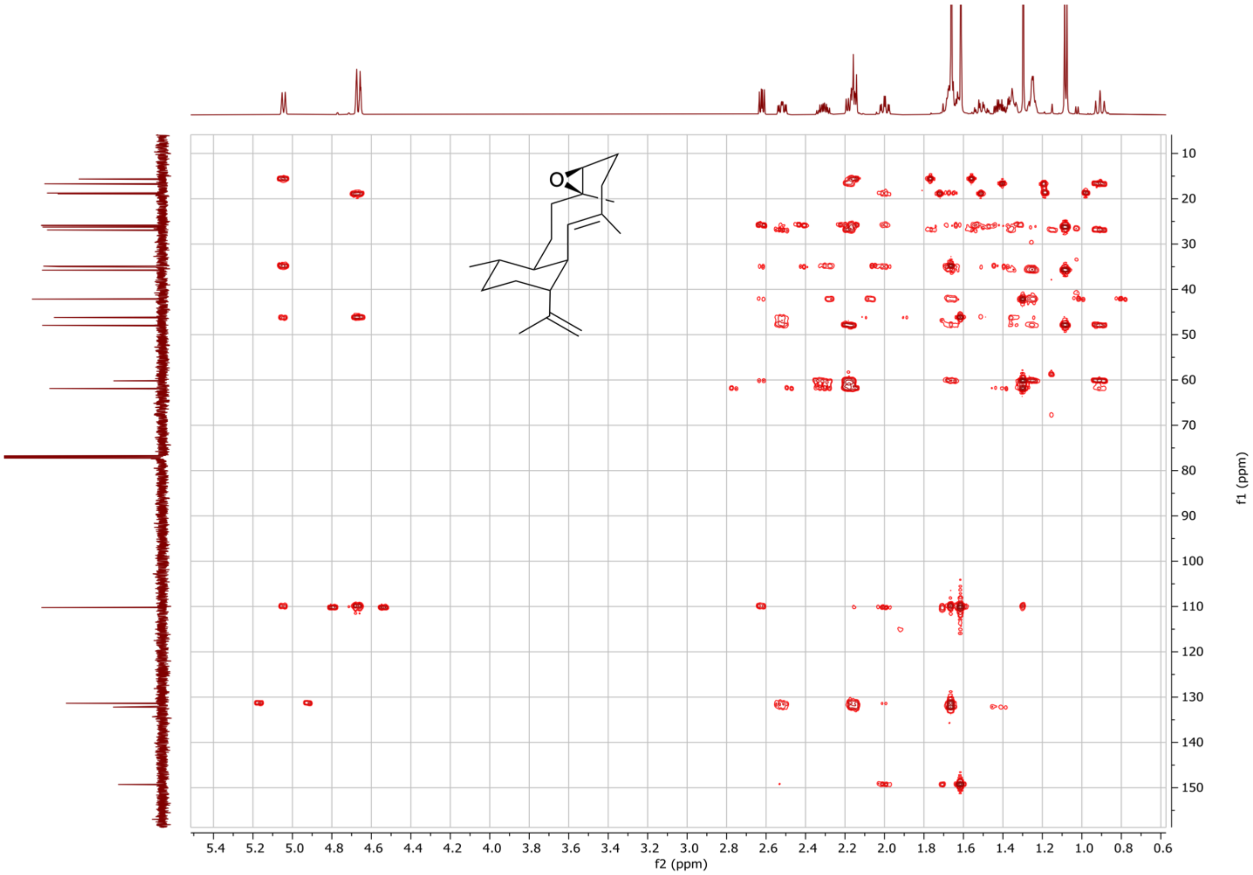

Figure S10. ^{1}H-^{1}H NOESY spectrum of **9** in chloroform-*d* (600 MHz).

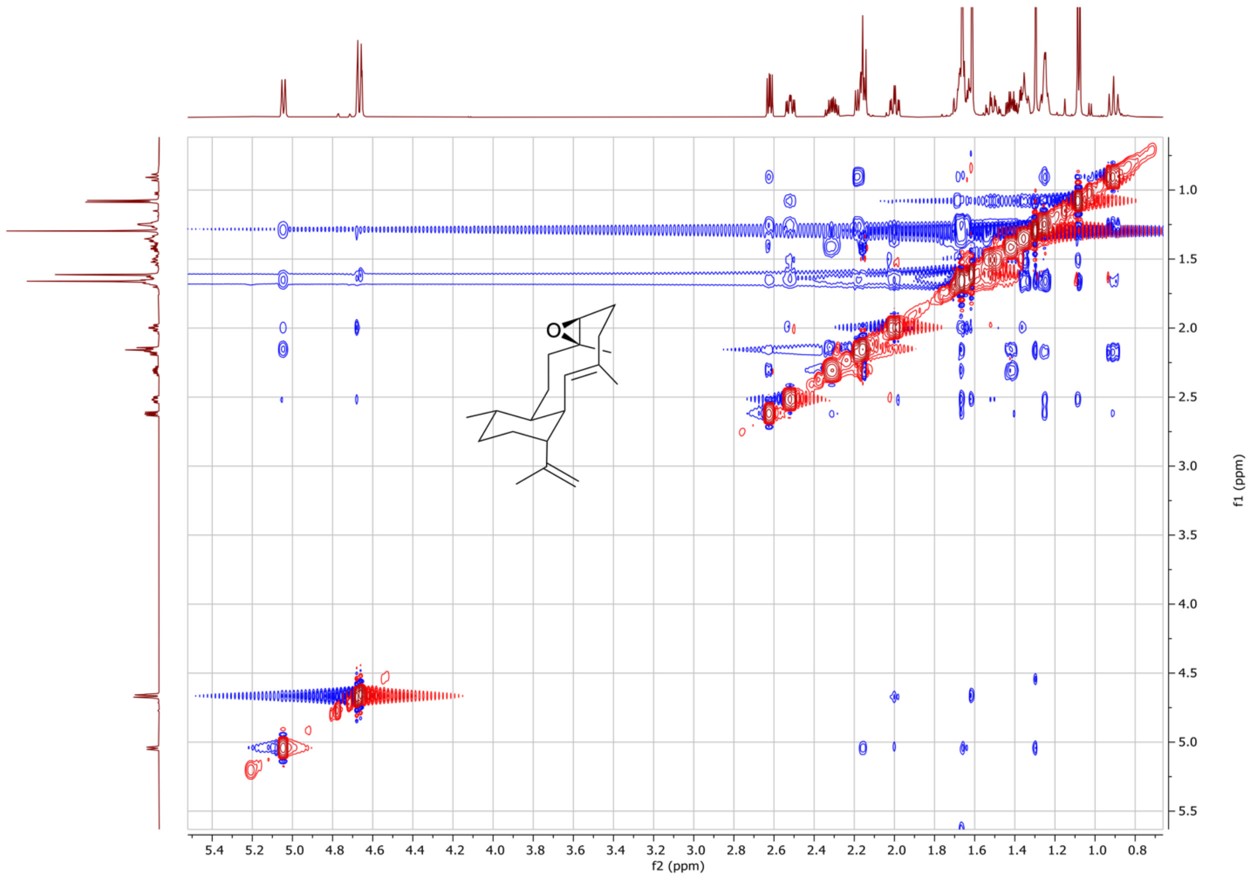

Figure S11. Relative free energy profiles for the Cope rearrangement in *cis*-eunicellanes. The 2*E*-*cis*-eunicellane **1** does not undergo observable thermal Cope rearrangement in our tested conditions (up to 200 °C for 5 h). (A) **1**-DD (lowest energy conformer) and (B) **1**-UD have higher activation barriers, whereas comparatively less stable conformer (C) **1** has the lower activation barrier.

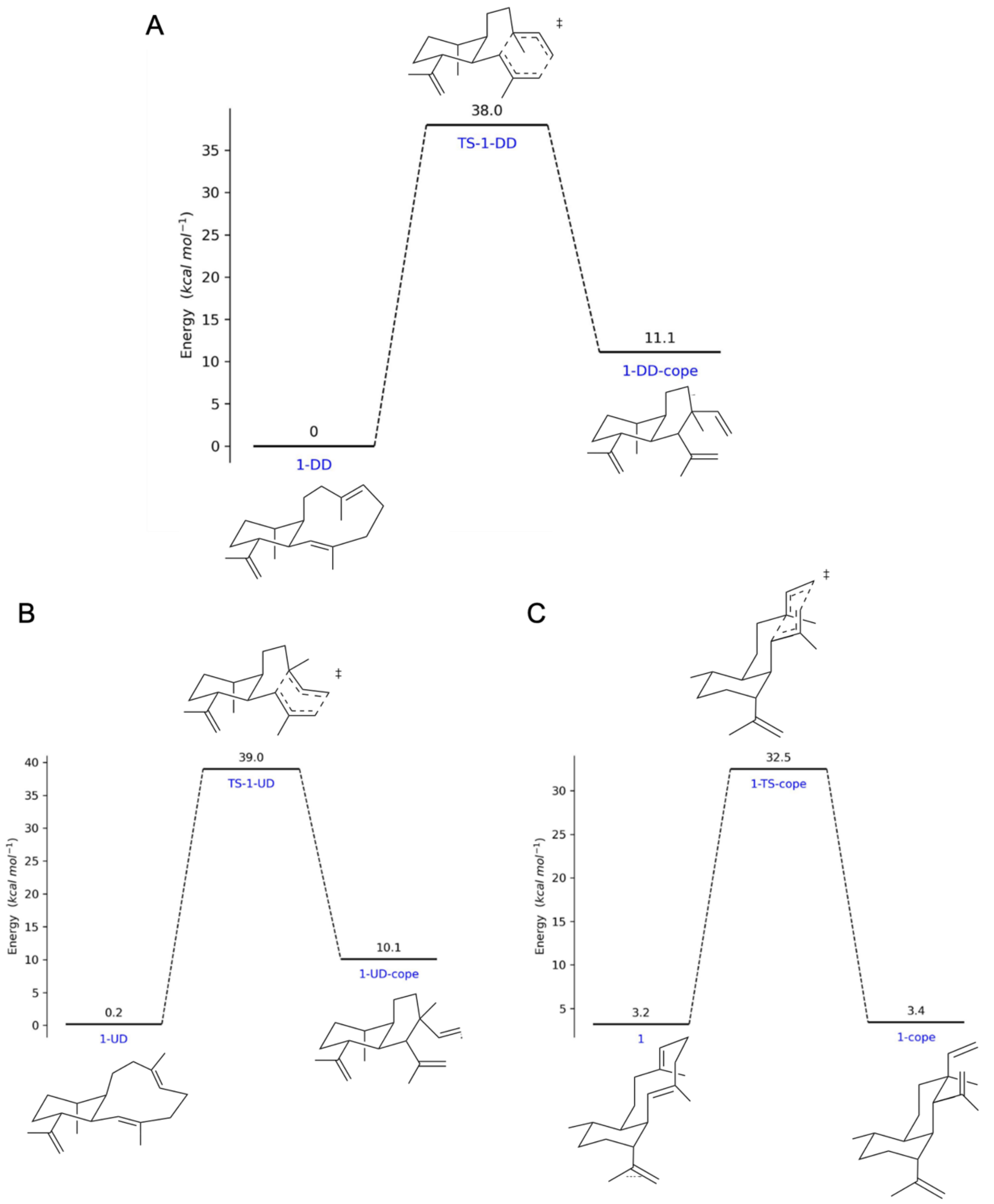

Figure S12. Relative free energy profiles for the oxy-Cope rearrangement in **11**. The free energies below were calculated using mPW1PW91/6–31+G(d/p)/SMD(chloroform). Similar calculations with water resulted in free energies of 0.0, 26.9, -2.8, and -13.8 for **11**, **11-TS**, **enol-12**, and **keto-12**, respectively.

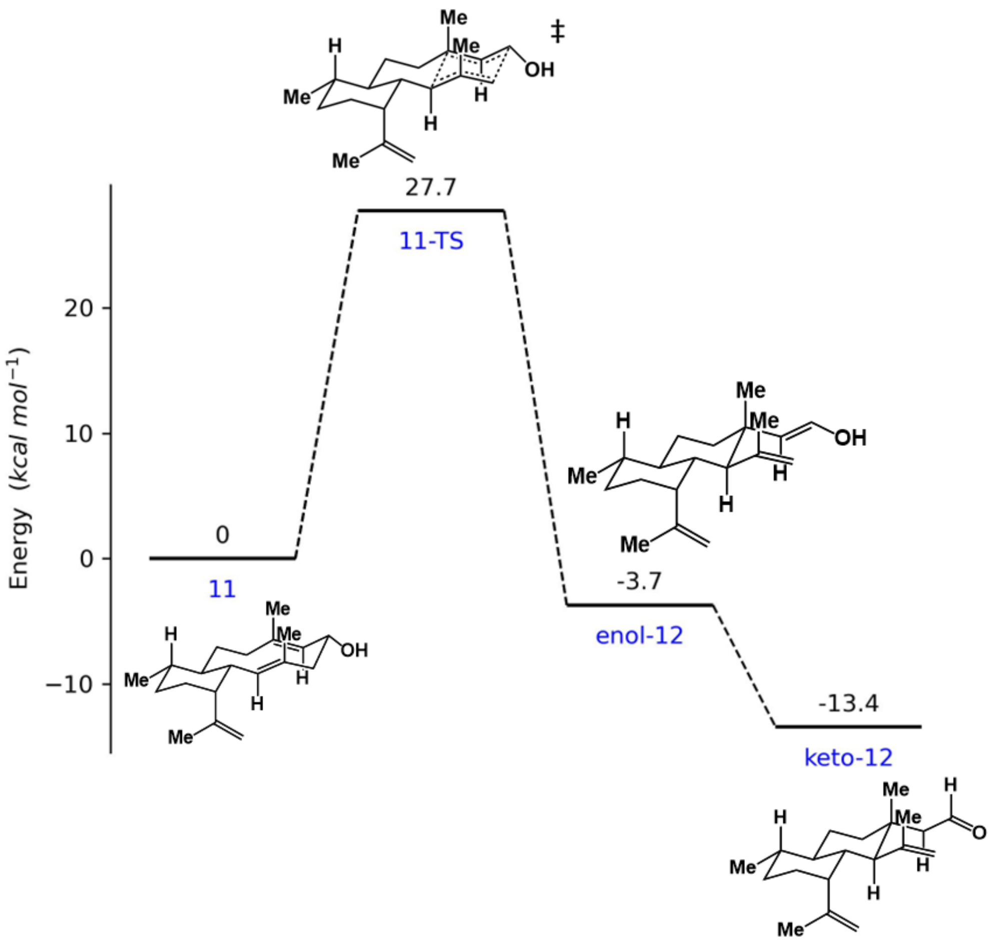

Figure S13. ^{1}H NMR spectrum of **14** in chloroform-d (600 MHz).

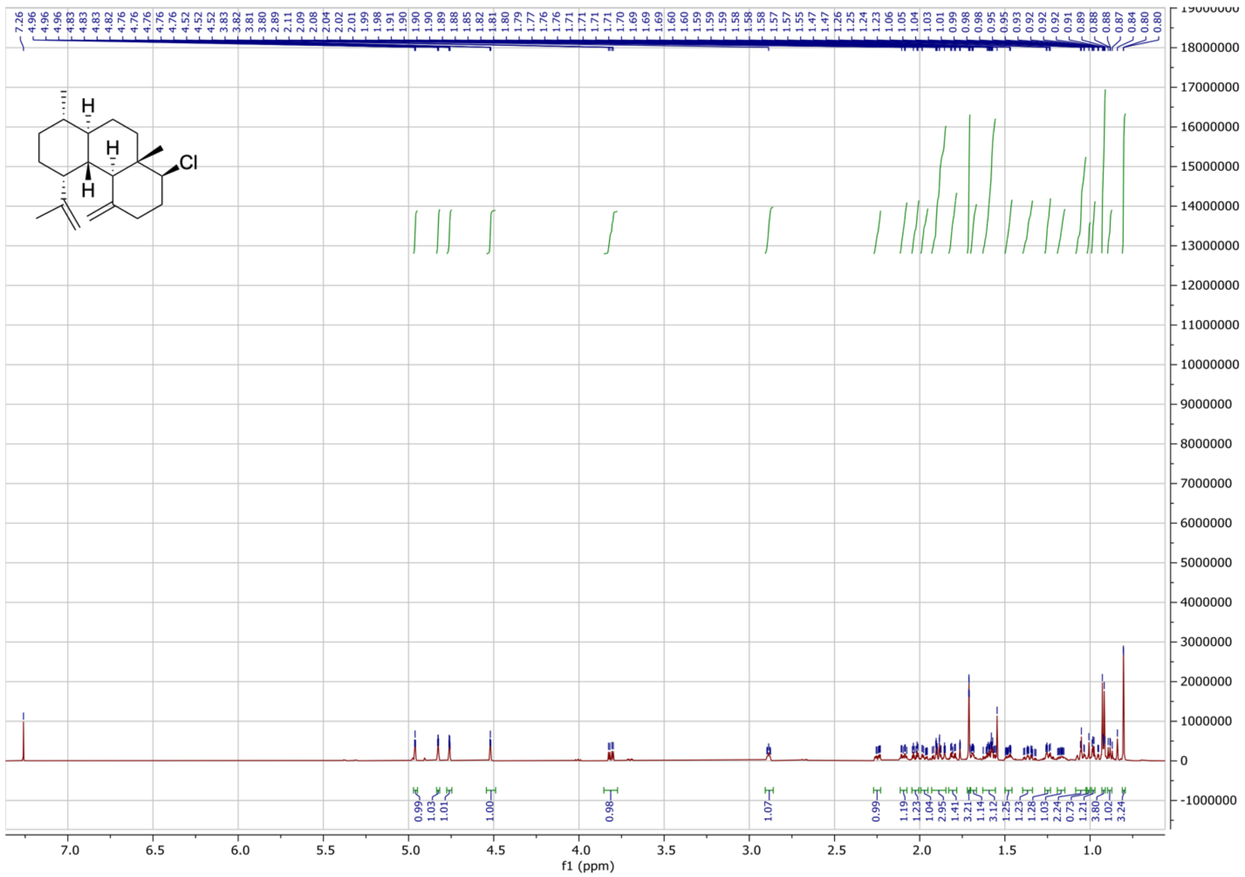

Figure S14. ^{13}C NMR spectrum of **14** in chloroform-*d* (151 MHz).

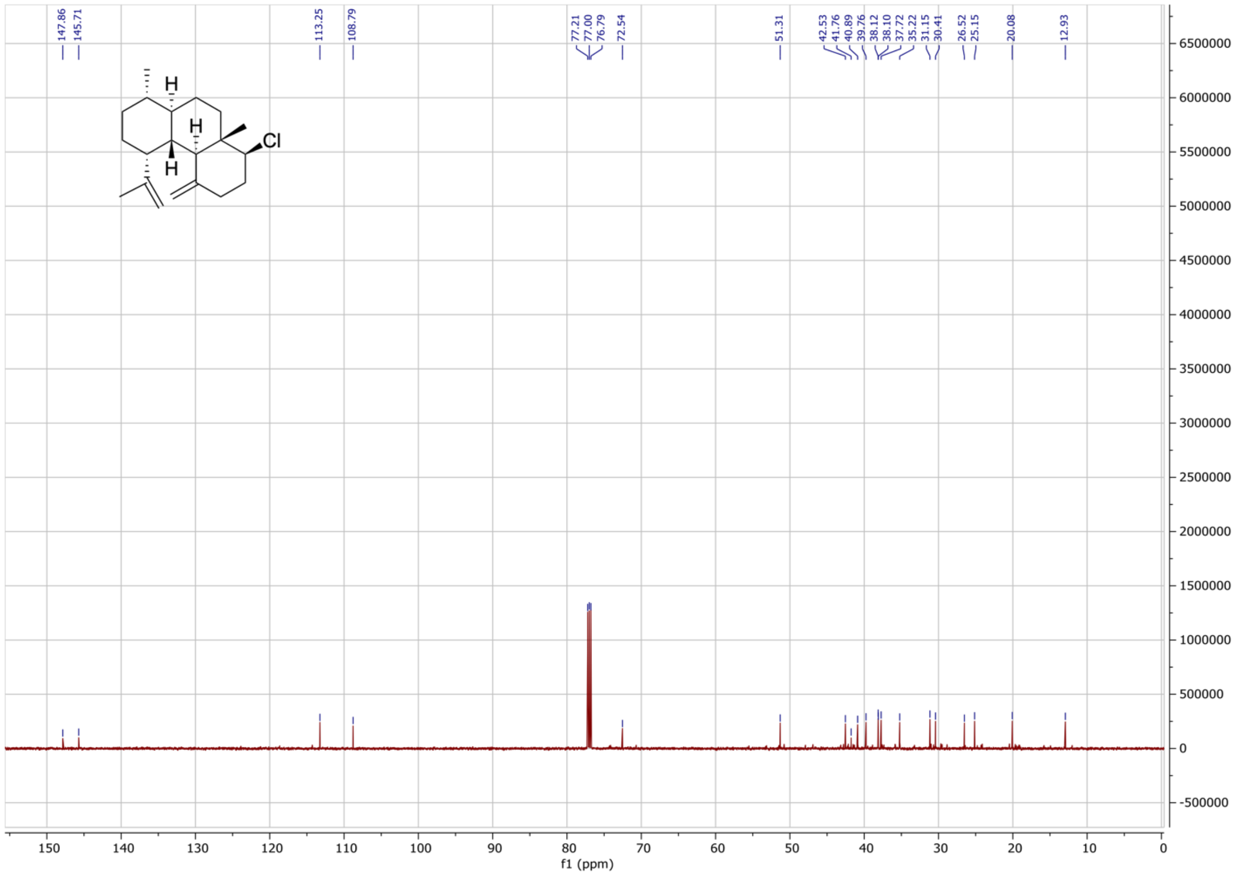

Figure S15. ^{1}H-^{1}H COSY spectrum of **14** in chloroform-*d* (600 MHz).

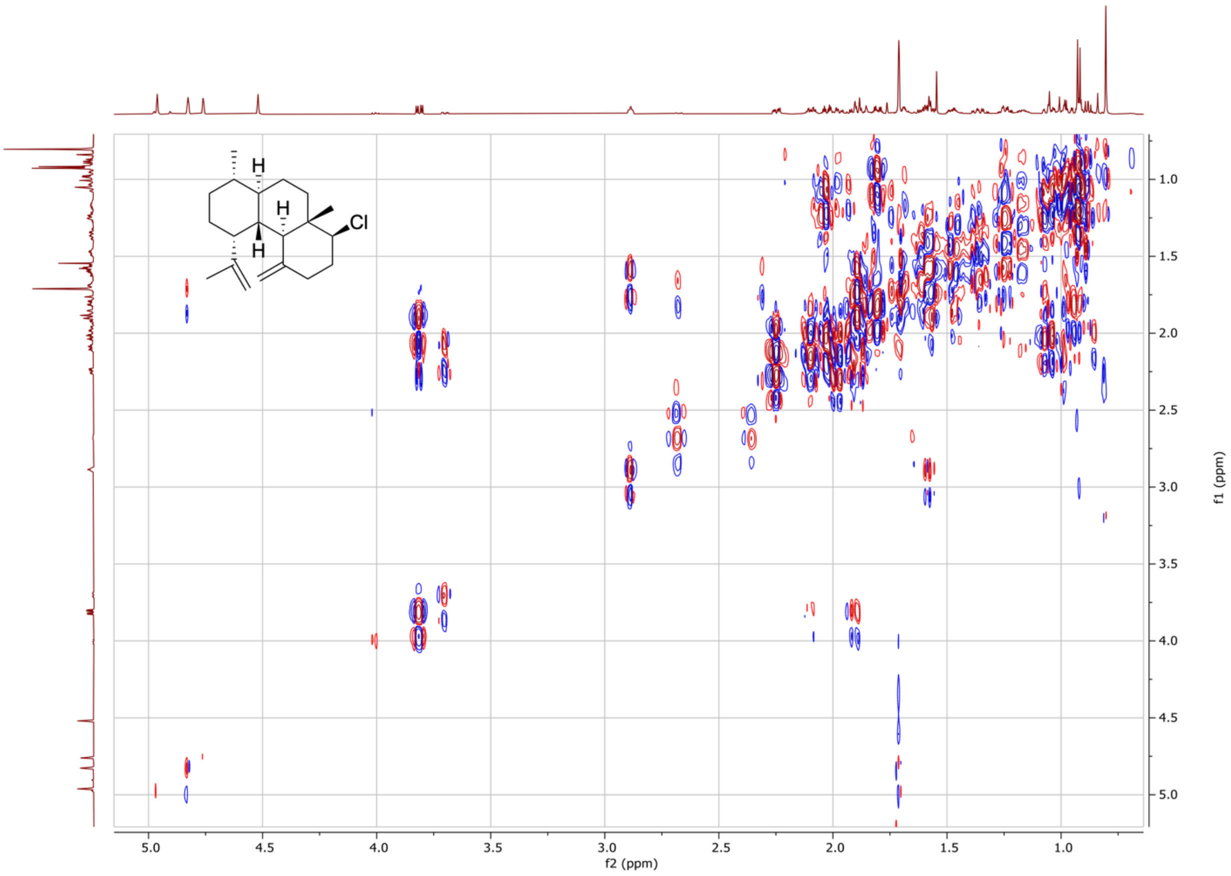

Figure S16. ^{1}H-^{13}C HSQC spectrum of **14** in chloroform-*d* (600 MHz).

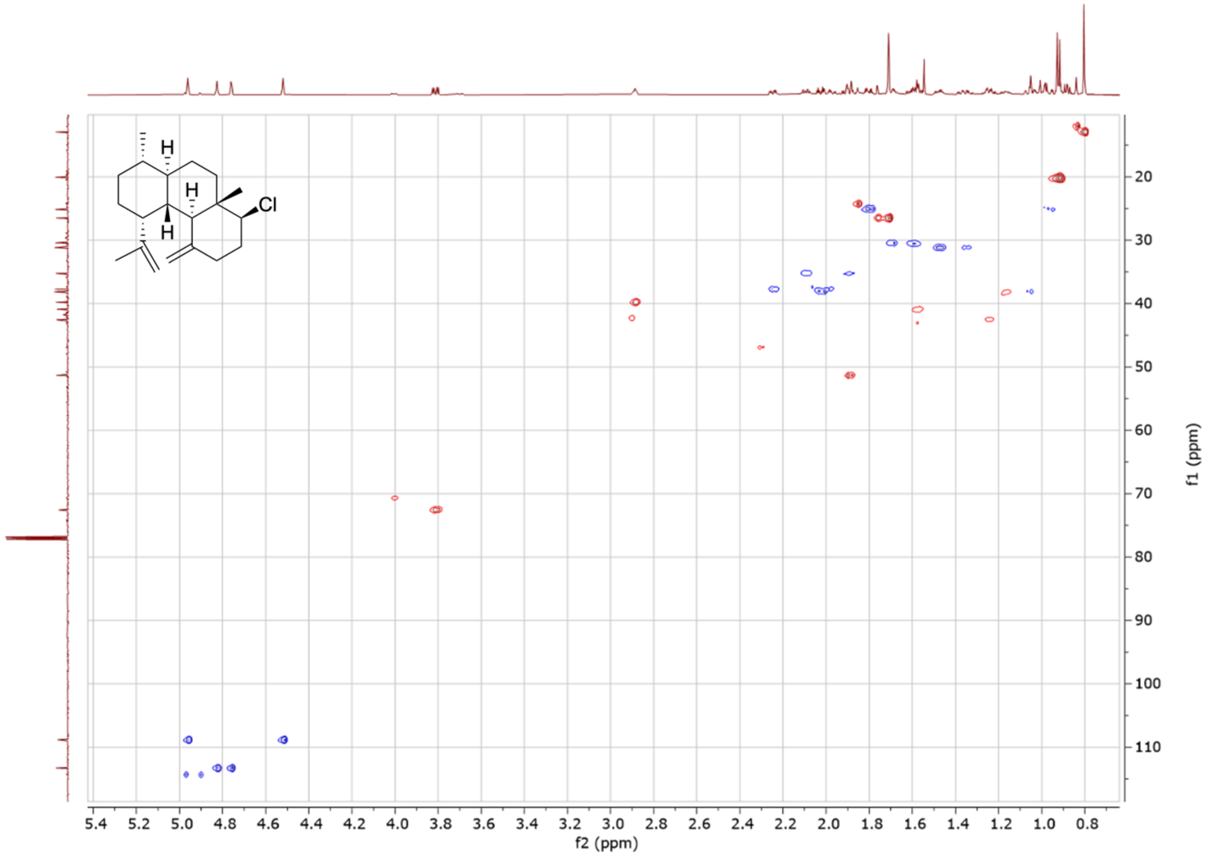

Figure S17. Selective 1D NOESY spectrum of **14** in chloroform-*d* with selective excitation at 3.816 ppm (H-6); mixing time = 300 ms.

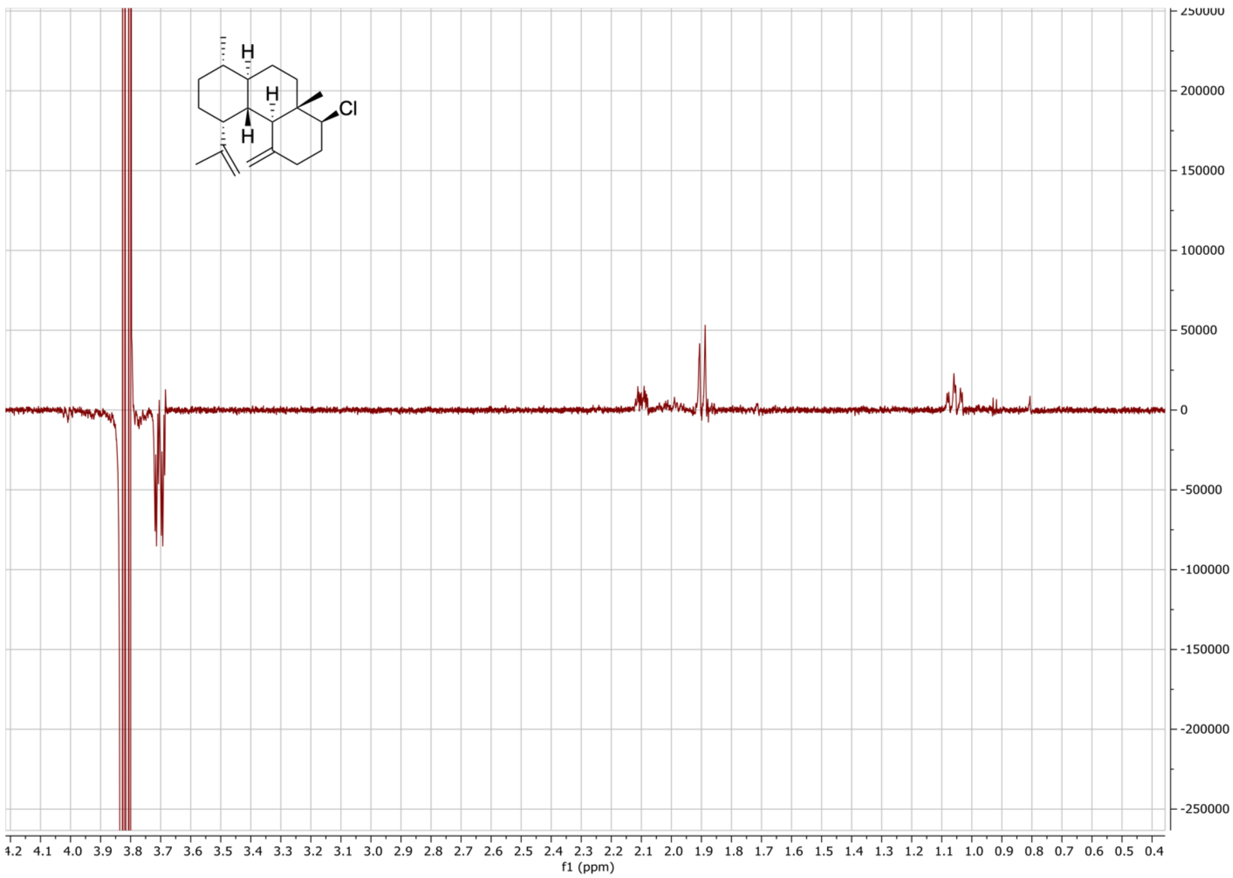

Figure S18. ^{1}H NMR spectrum of **15** in chloroform-*d* (600 MHz).

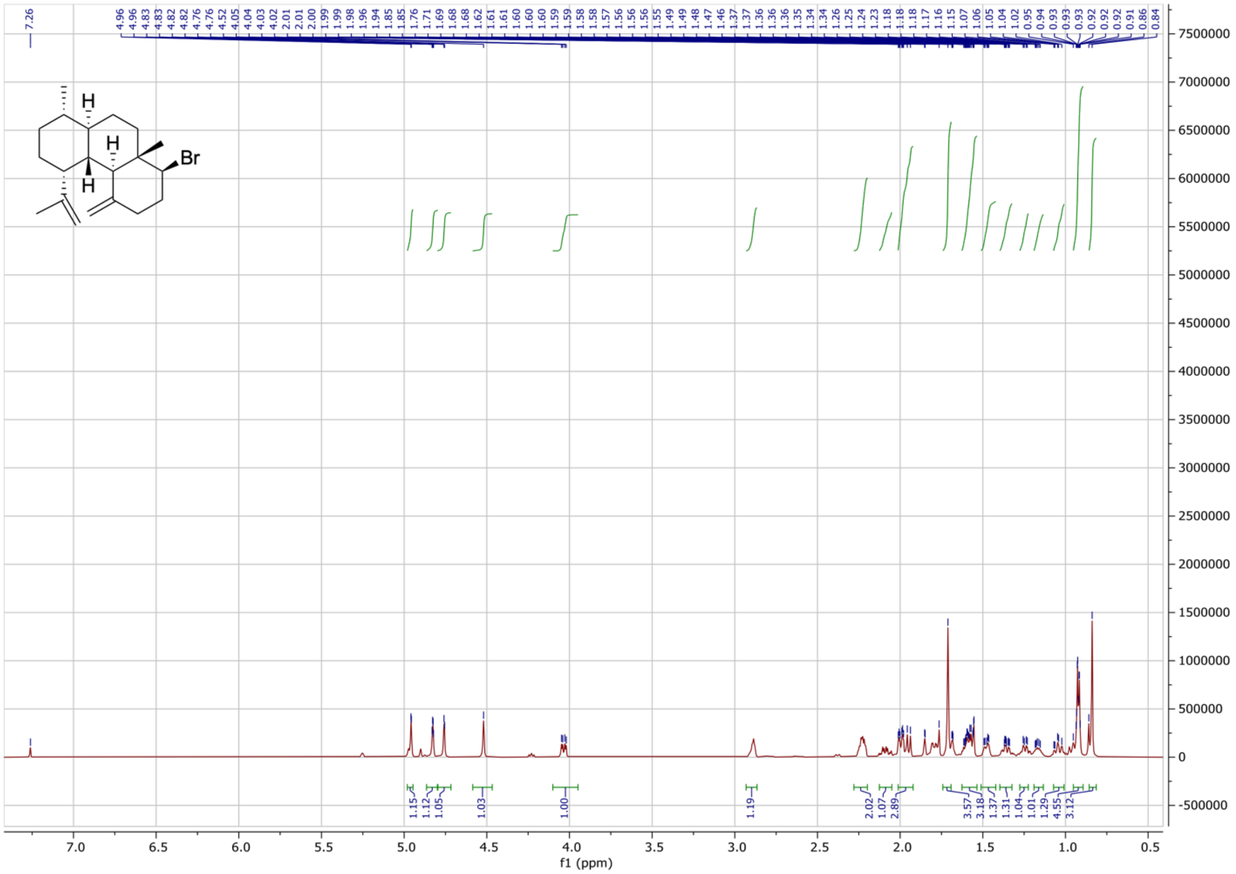

Figure S19. ^{13}C NMR spectrum of **15** in chloroform-*d* (151 MHz).

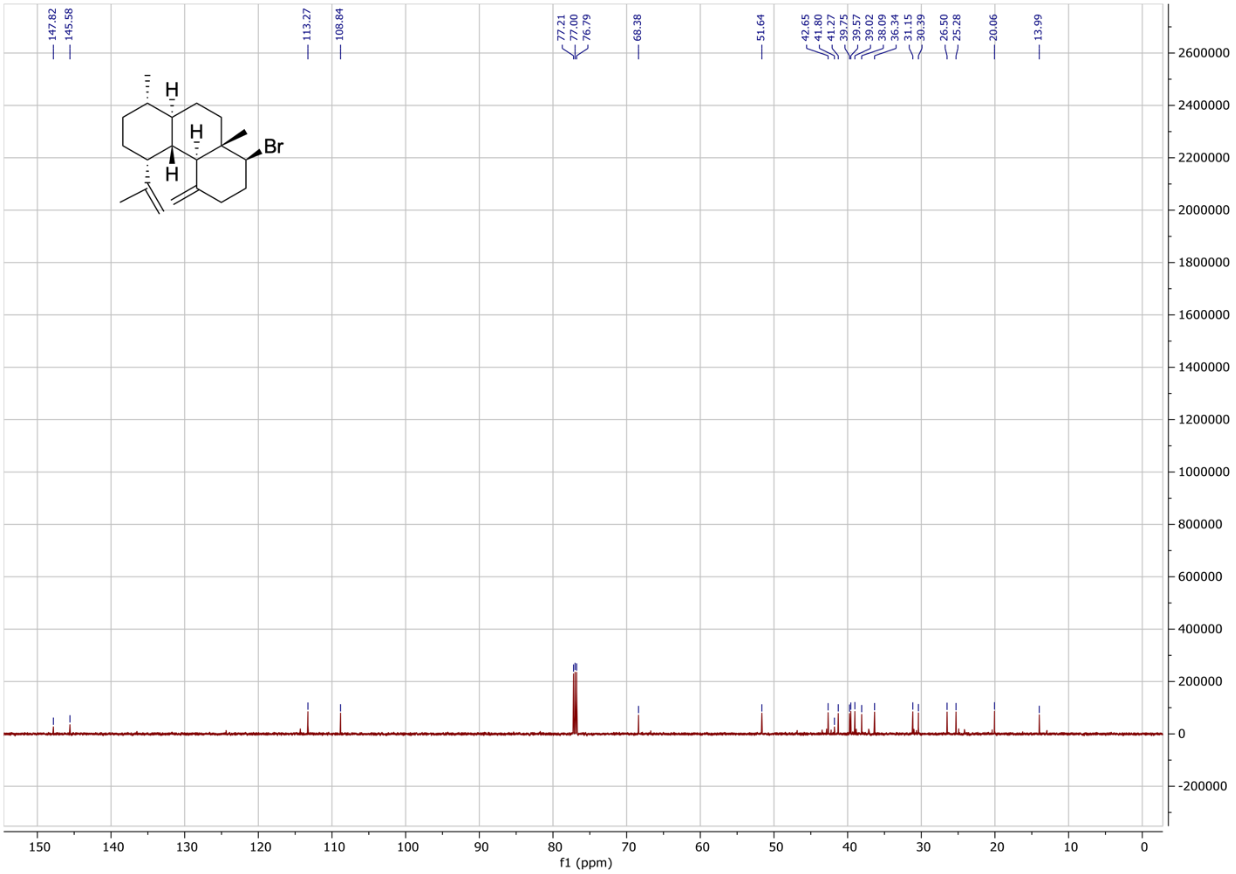

Figure S20. ^{1}H-^{1}H COSY spectrum of **15** in chloroform-*d* (600 MHz).

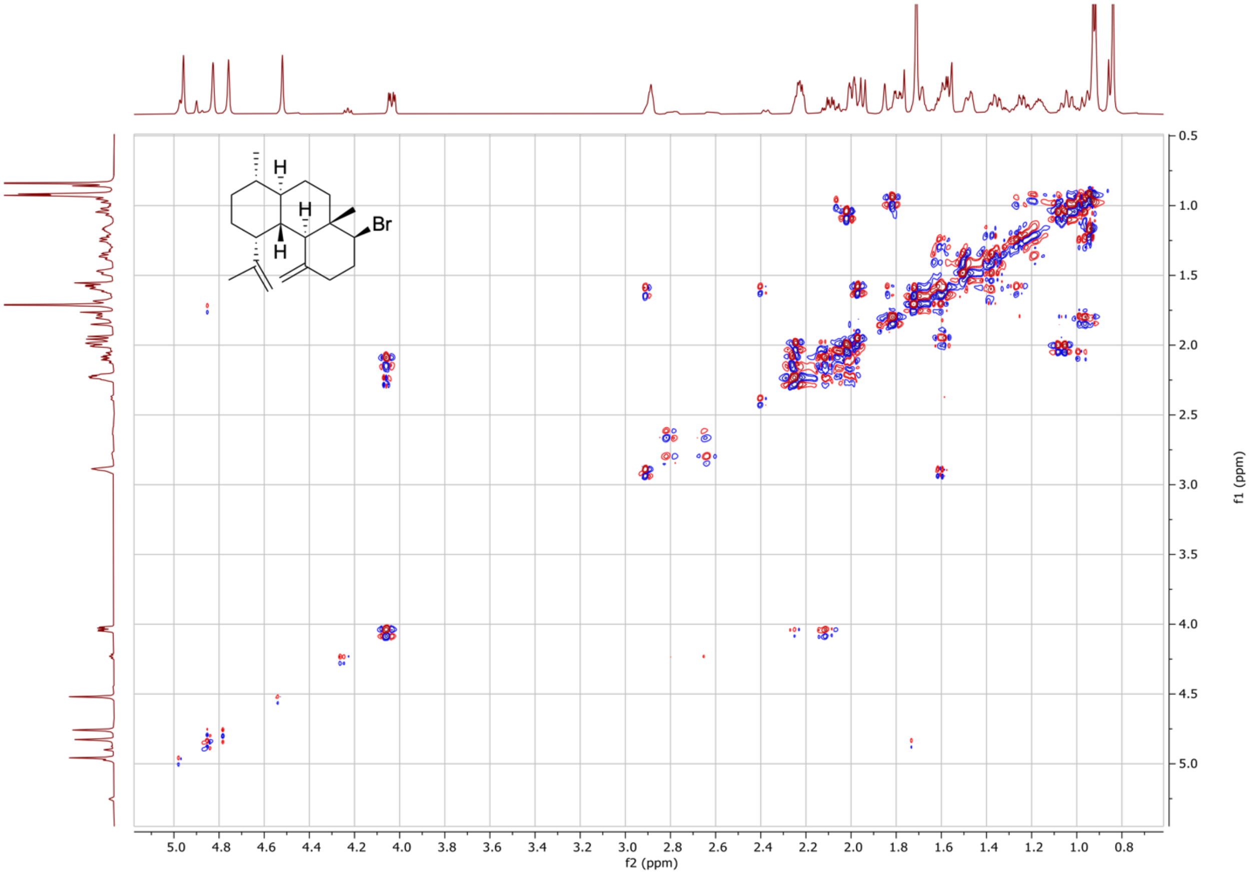

Figure S21. ^{1}H-^{13}C HSQC spectrum of **15** in chloroform-*d* (600 MHz).

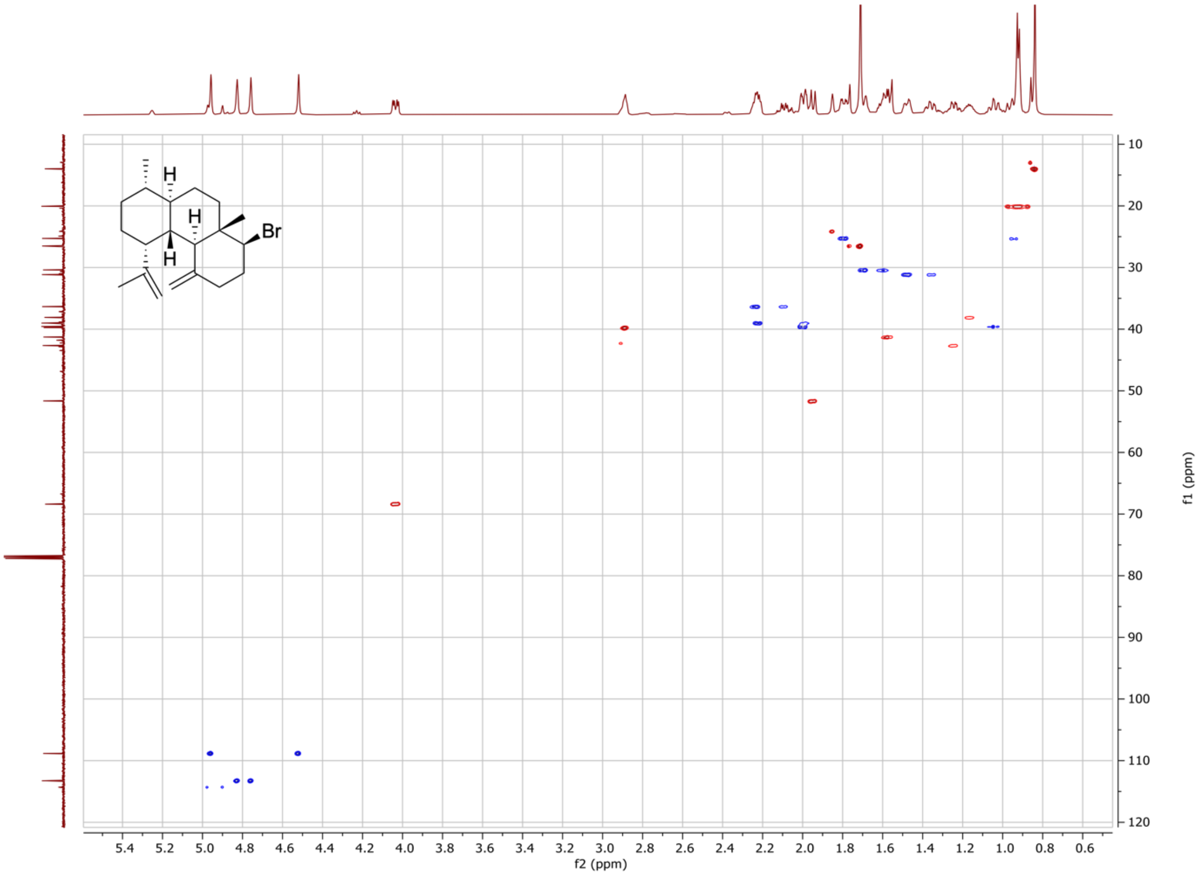

Figure S22. ^{1}H-^{13}C HMBC spectrum of **15** in chloroform-*d* (600 MHz).

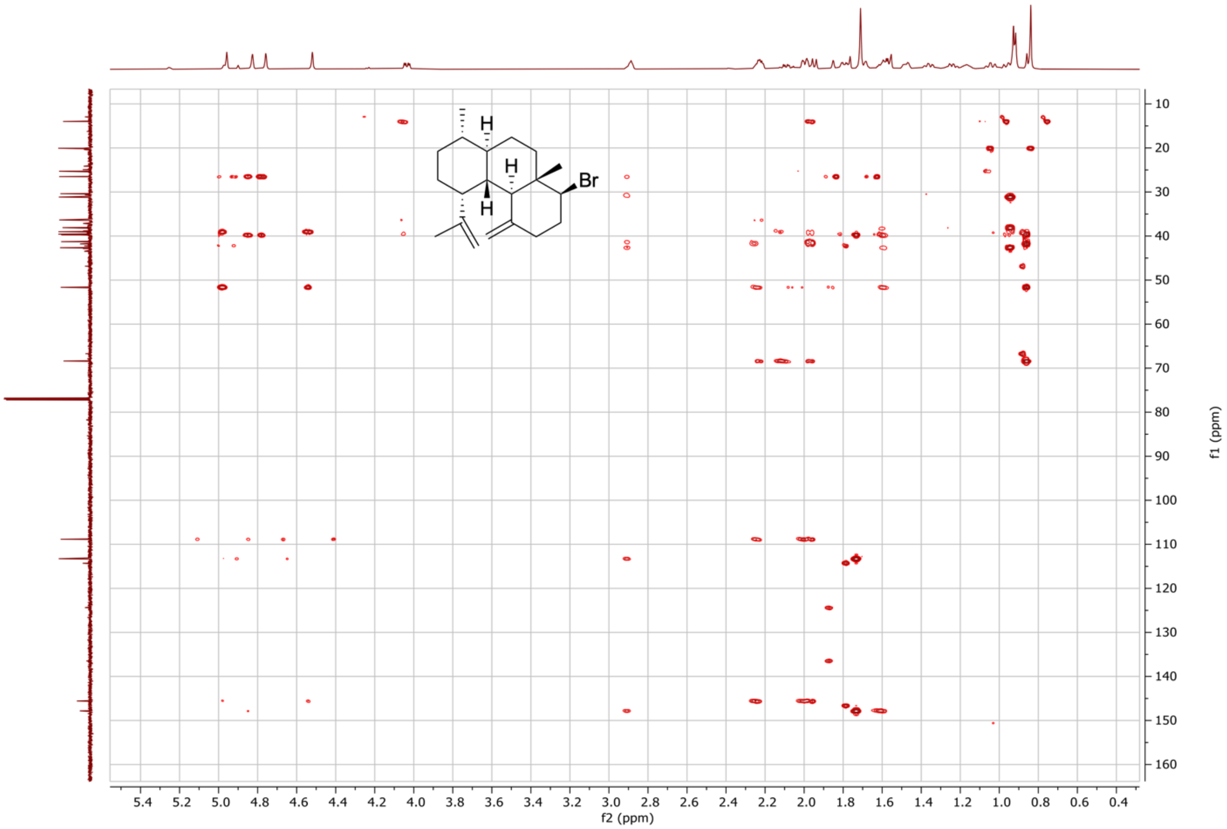

Figure S23. Selective 1D NOESY spectrum of **15** in chloroform-*d* with selective excitation at 4.04 ppm (H-6); mixing time = 300 ms.

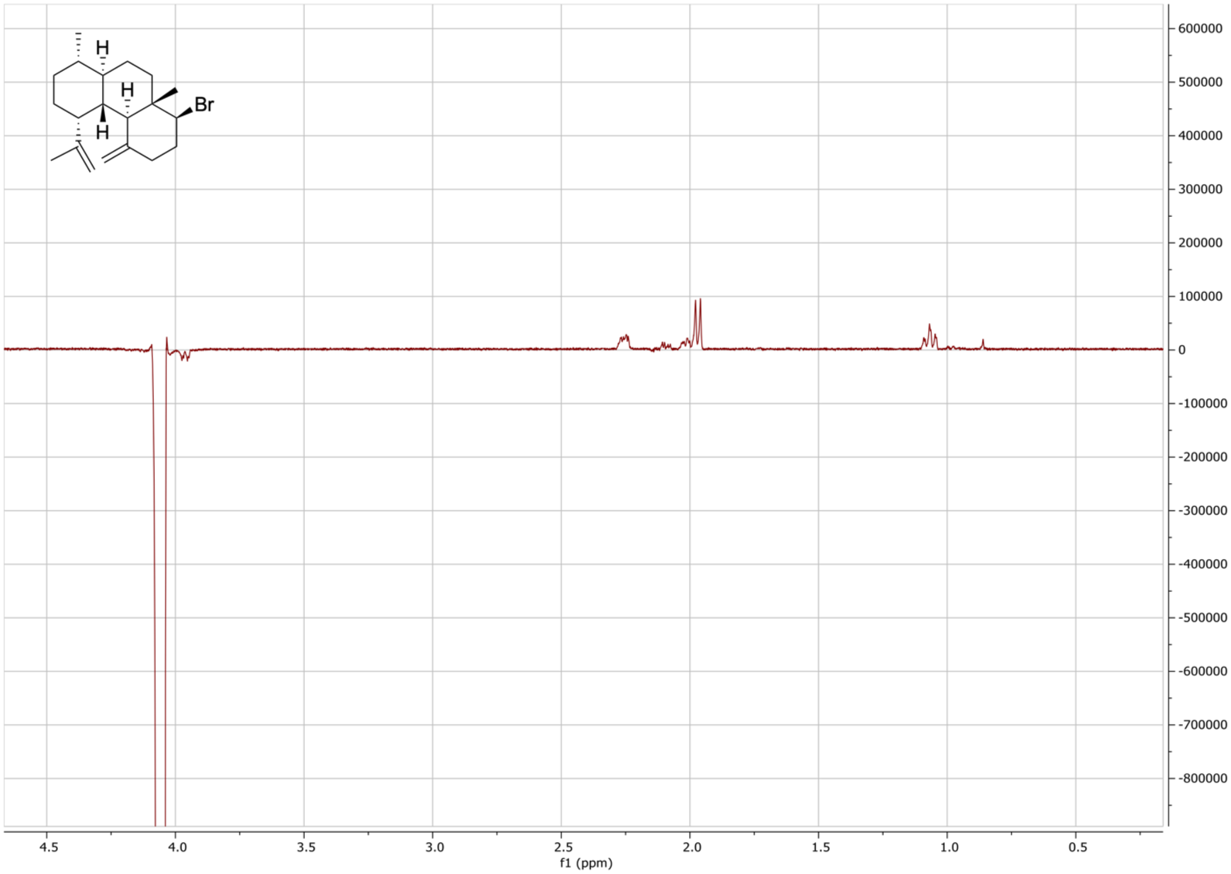

Figure S24. ^{1}H NMR spectrum of **16** in chloroform-*d* (600 MHz).

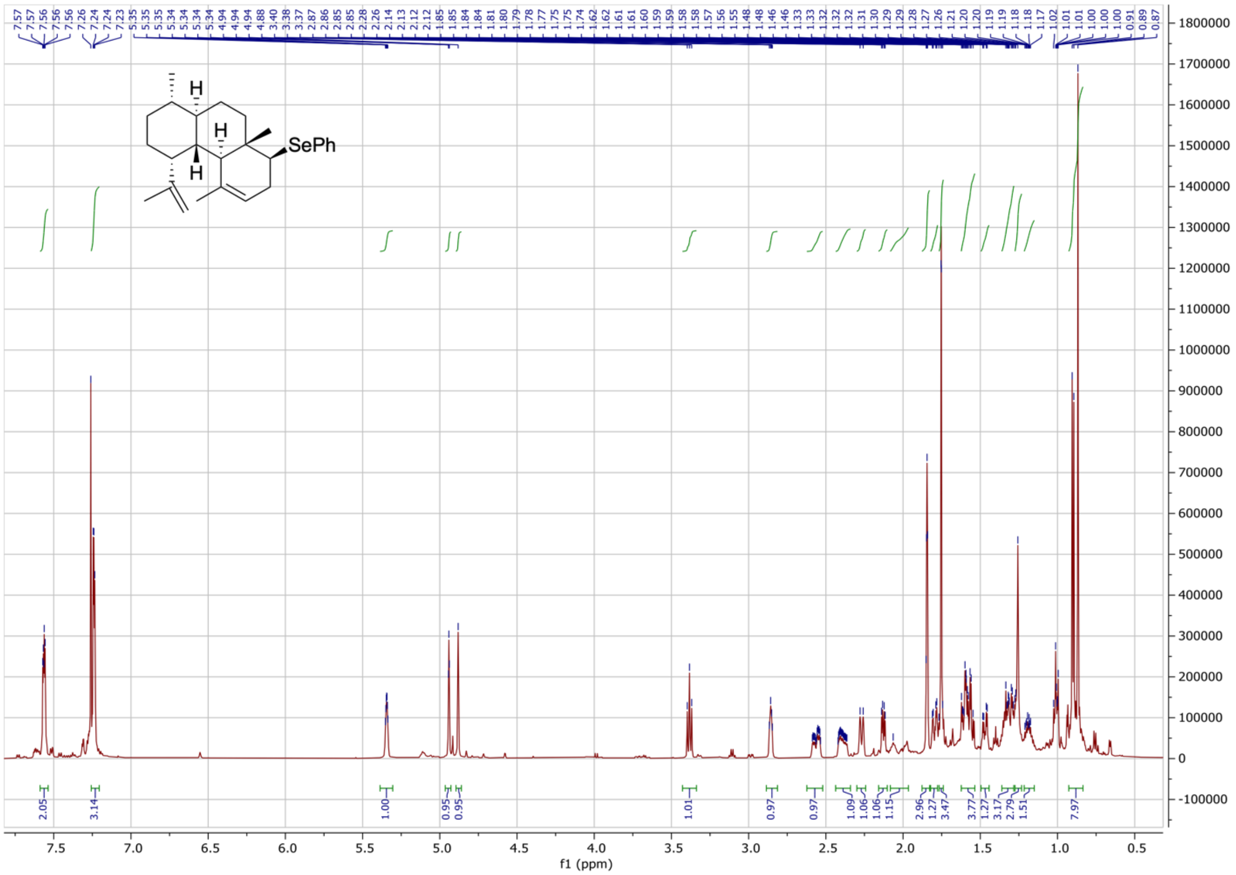

Figure S25. ^{13}C NMR spectrum of **16** in chloroform-*d* (151 MHz).

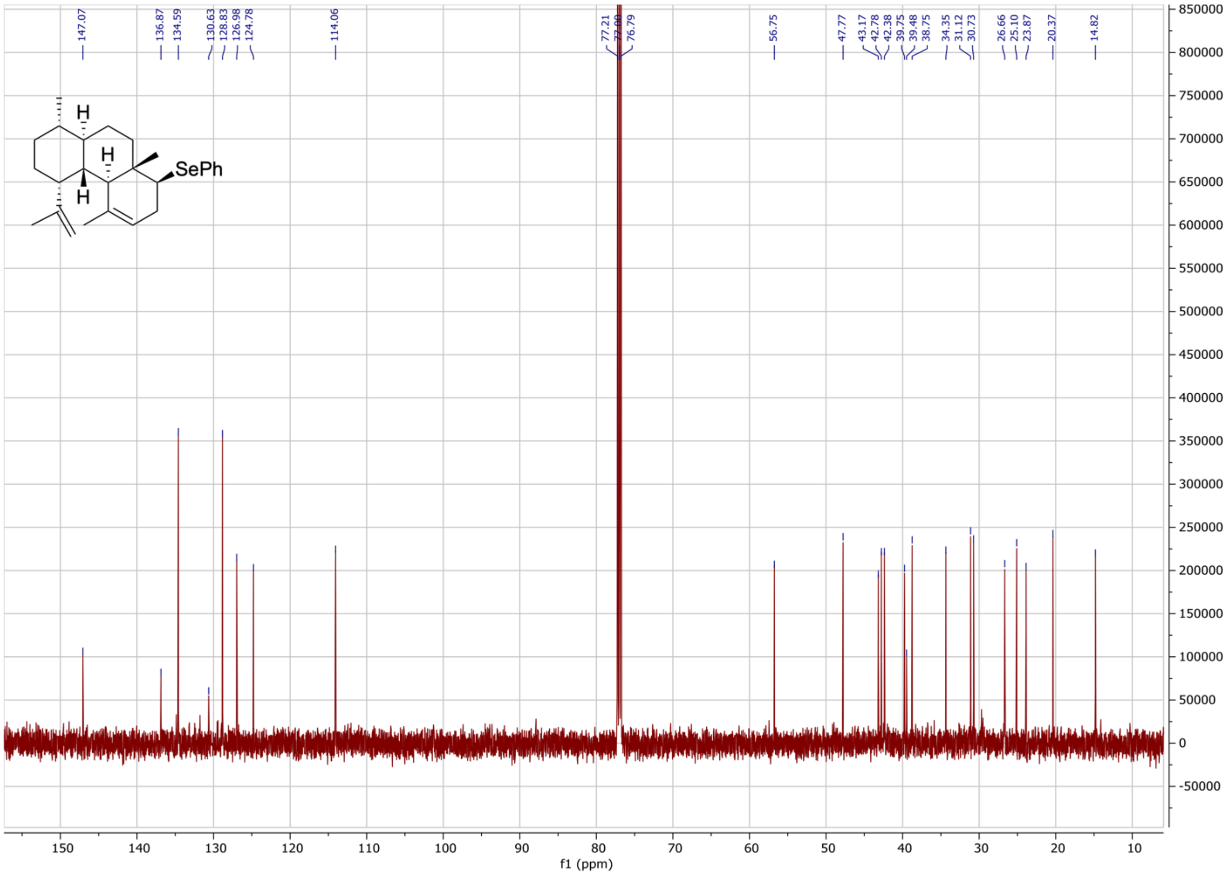

Figure S26. ^{1}H-^{1}H COSY spectrum of **16** in chloroform-*d* (600 MHz).

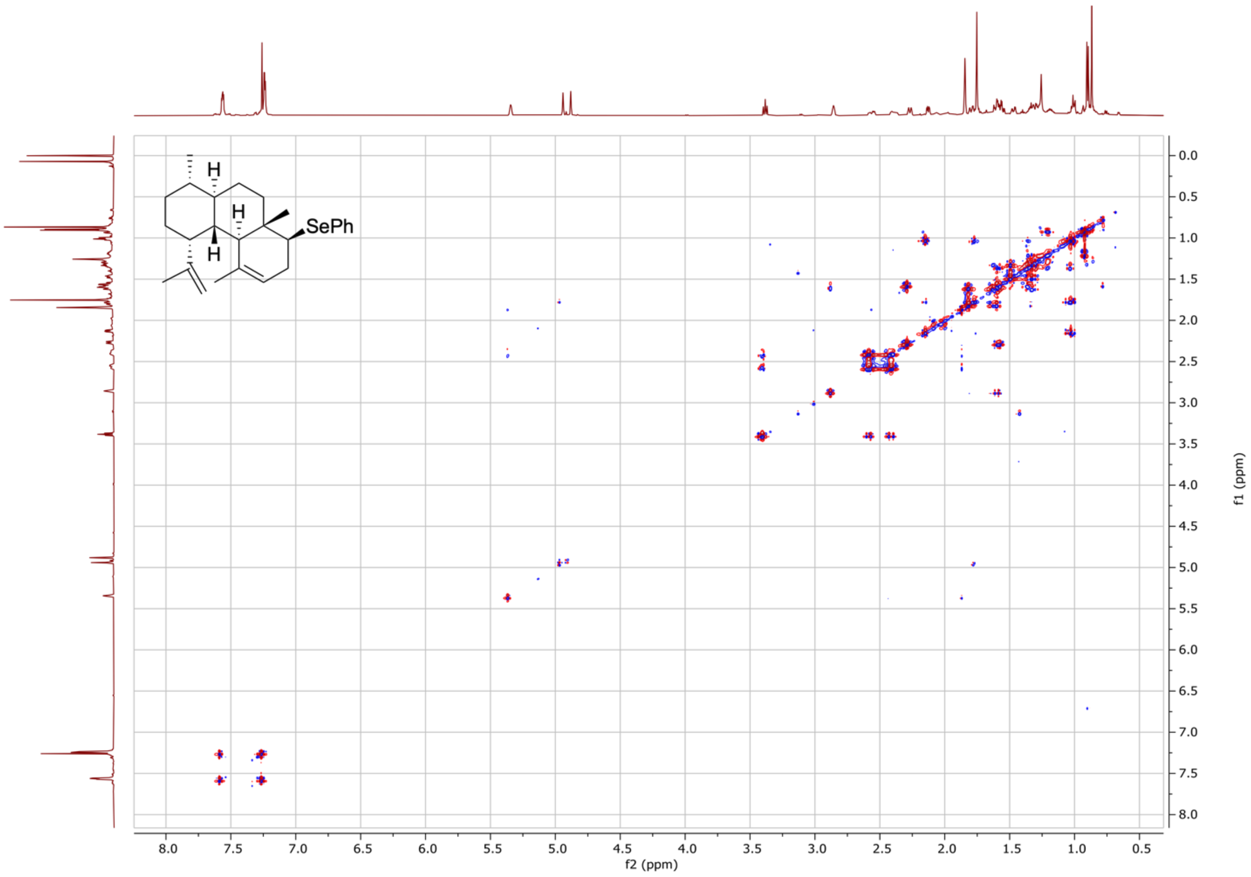

Figure S27. ^{1}H-^{13}C HSQC spectrum of **16** in chloroform-*d* (600 MHz).

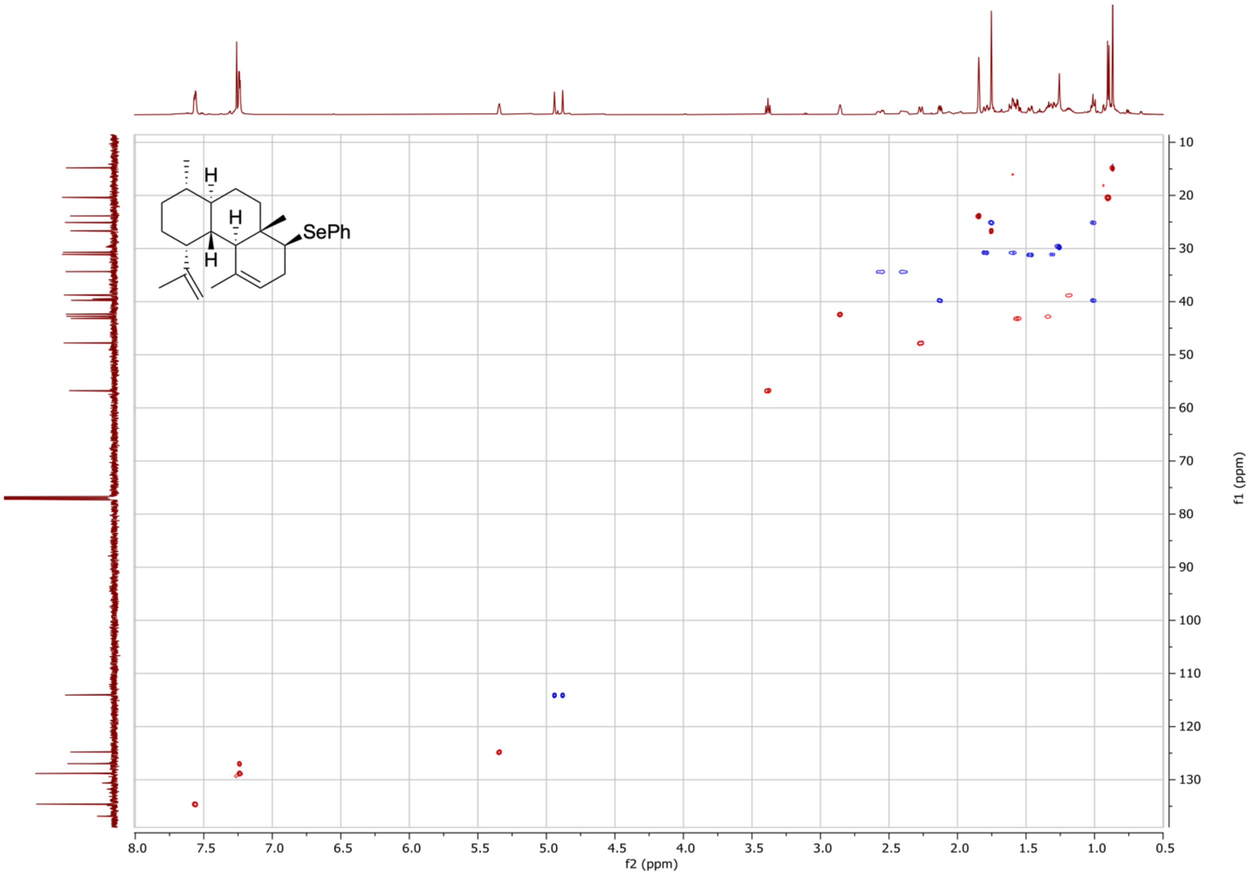

Figure S28. ^{1}H-^{13}C HMBC spectrum of **16** in chloroform-*d* (600 MHz).

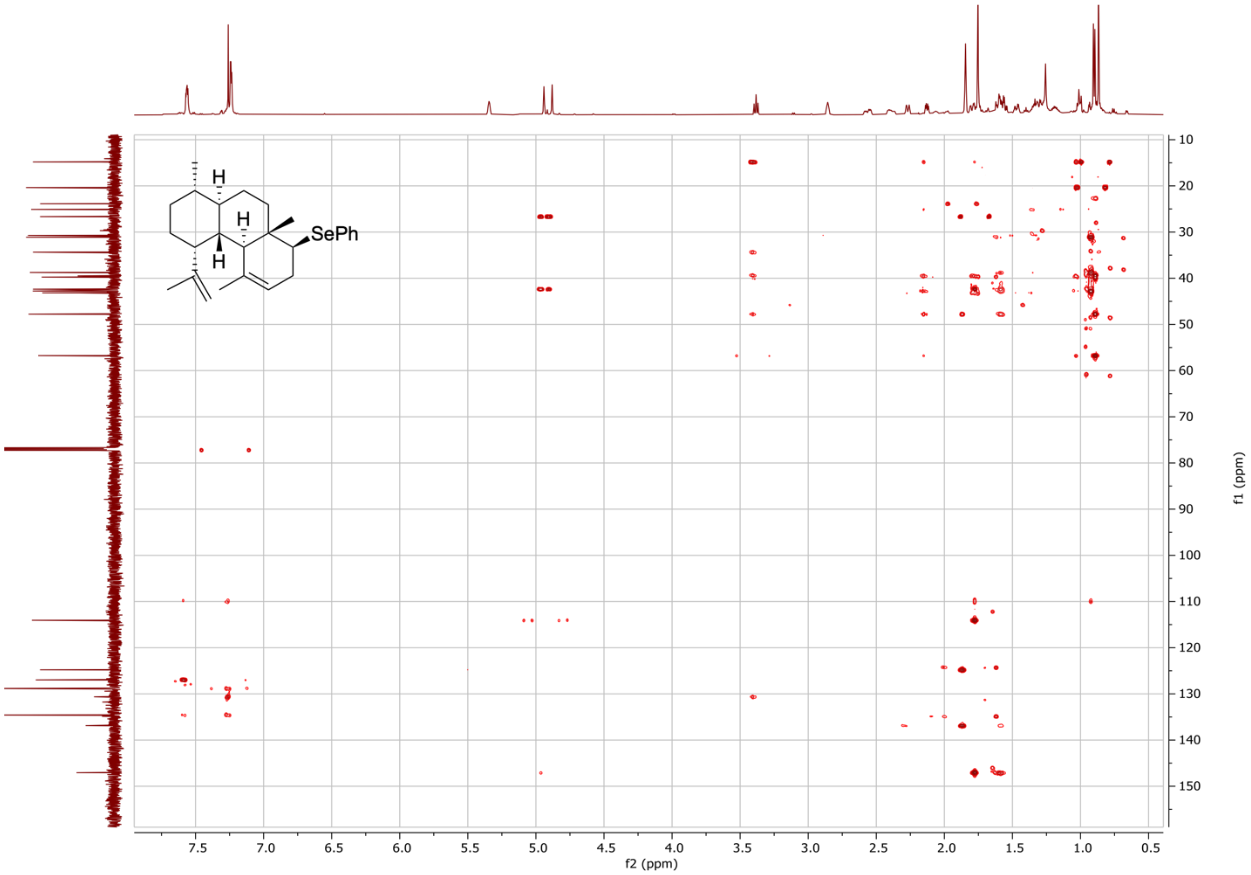

Figure S29. Selective 1D NOESY spectrum of **16** in chloroform-*d* with selective excitation at 3.38 ppm (H-6); mixing time = 300 ms.

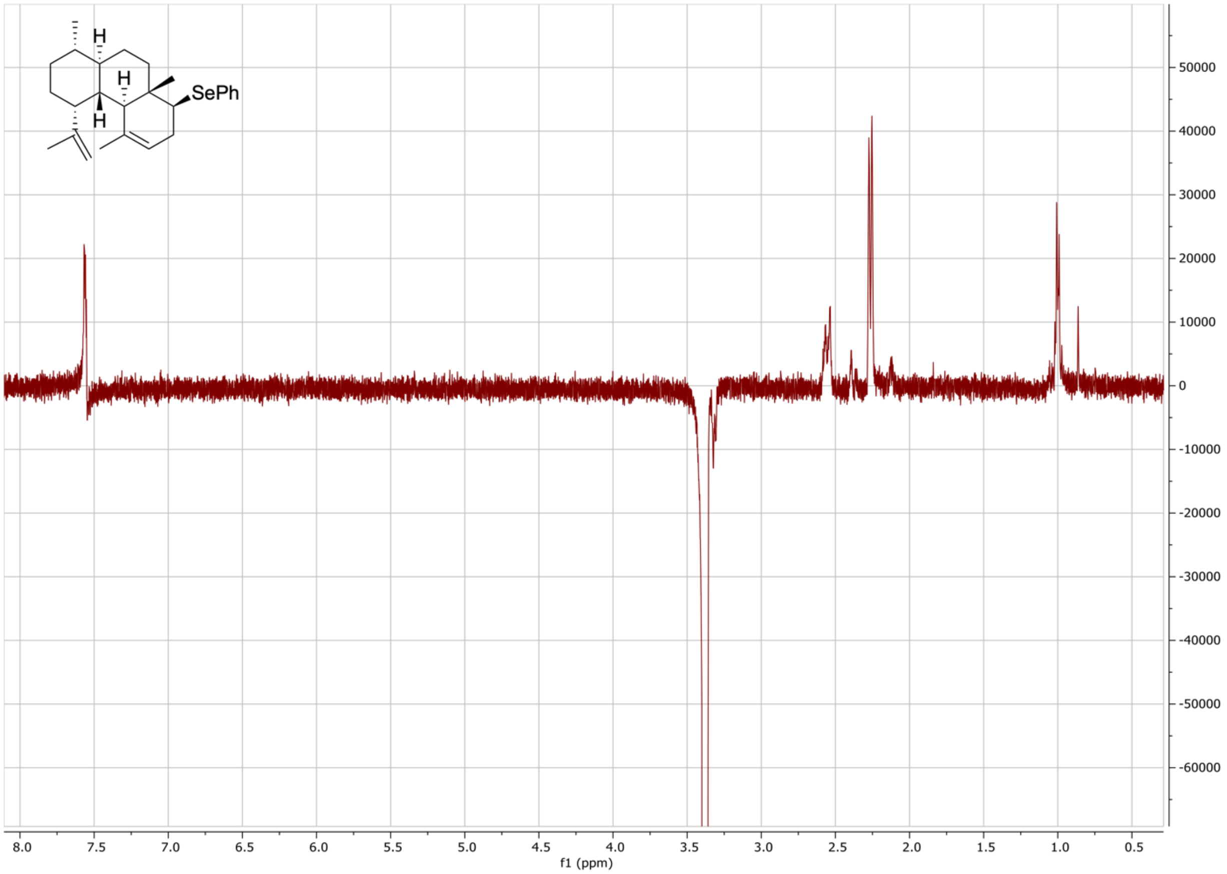

Figure S30. ^{1}H NMR spectrum of **17** in chloroform-*d* (600 MHz).

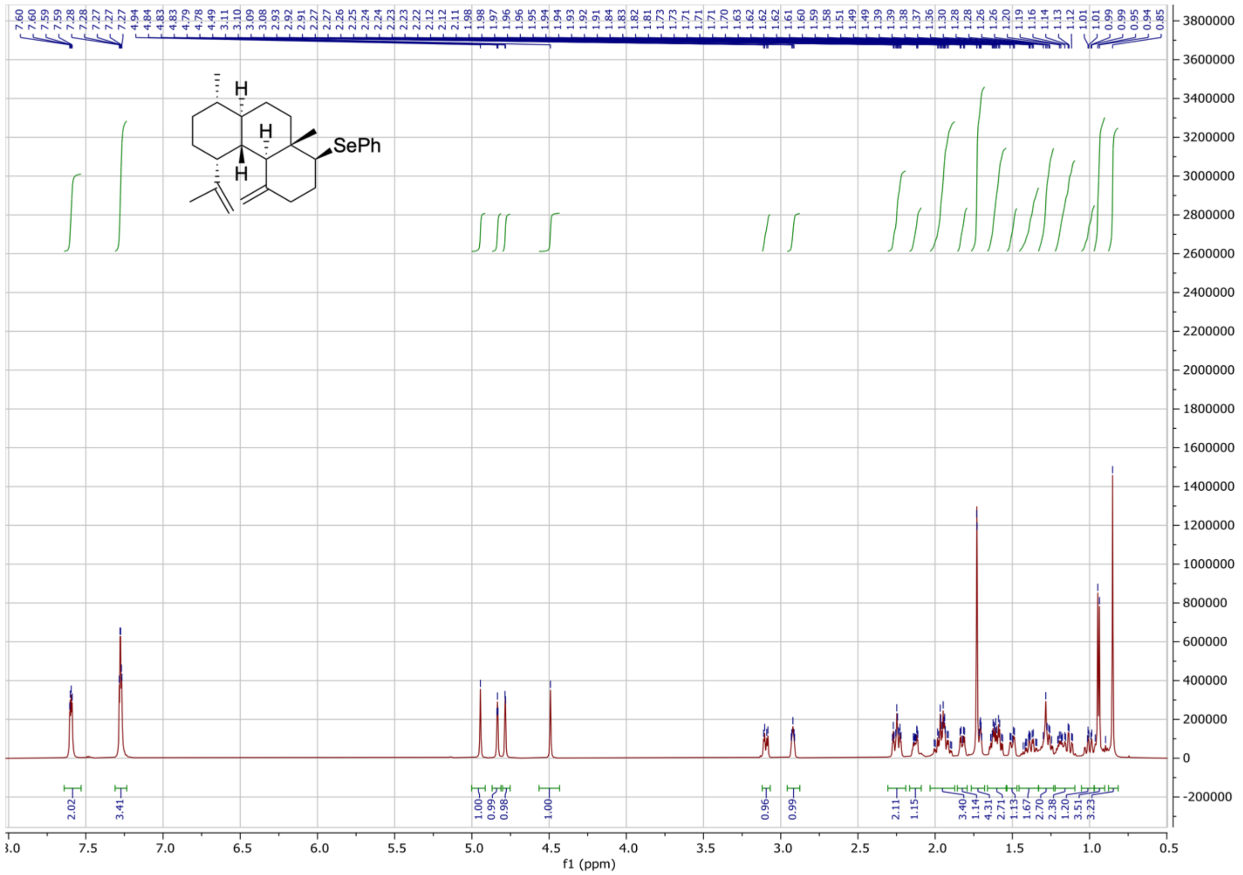

Figure S31. ^{13}C NMR spectrum of **17** in chloroform-*d* (151 MHz).

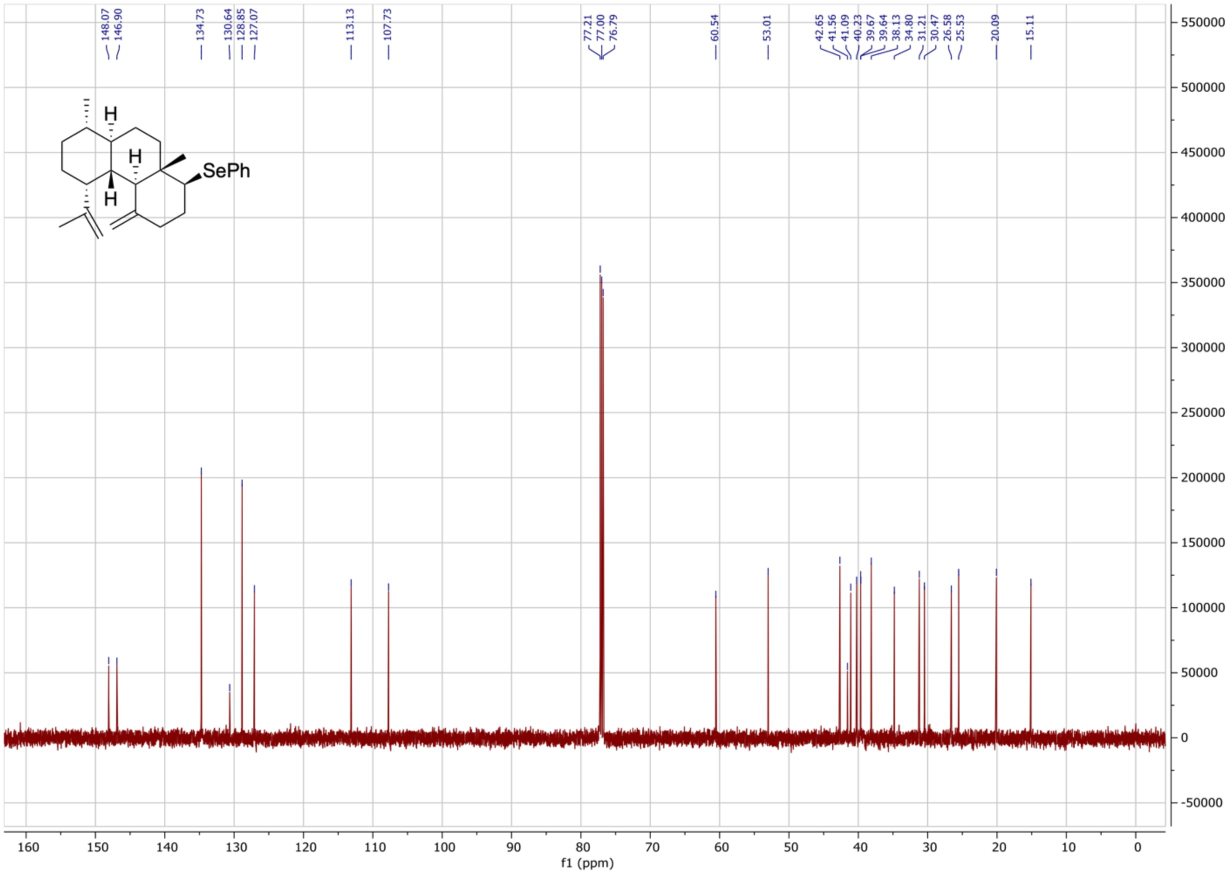

Figure S32. ^{1}H-^{1}H COSY spectrum of **17** in chloroform-*d* (600 MHz).

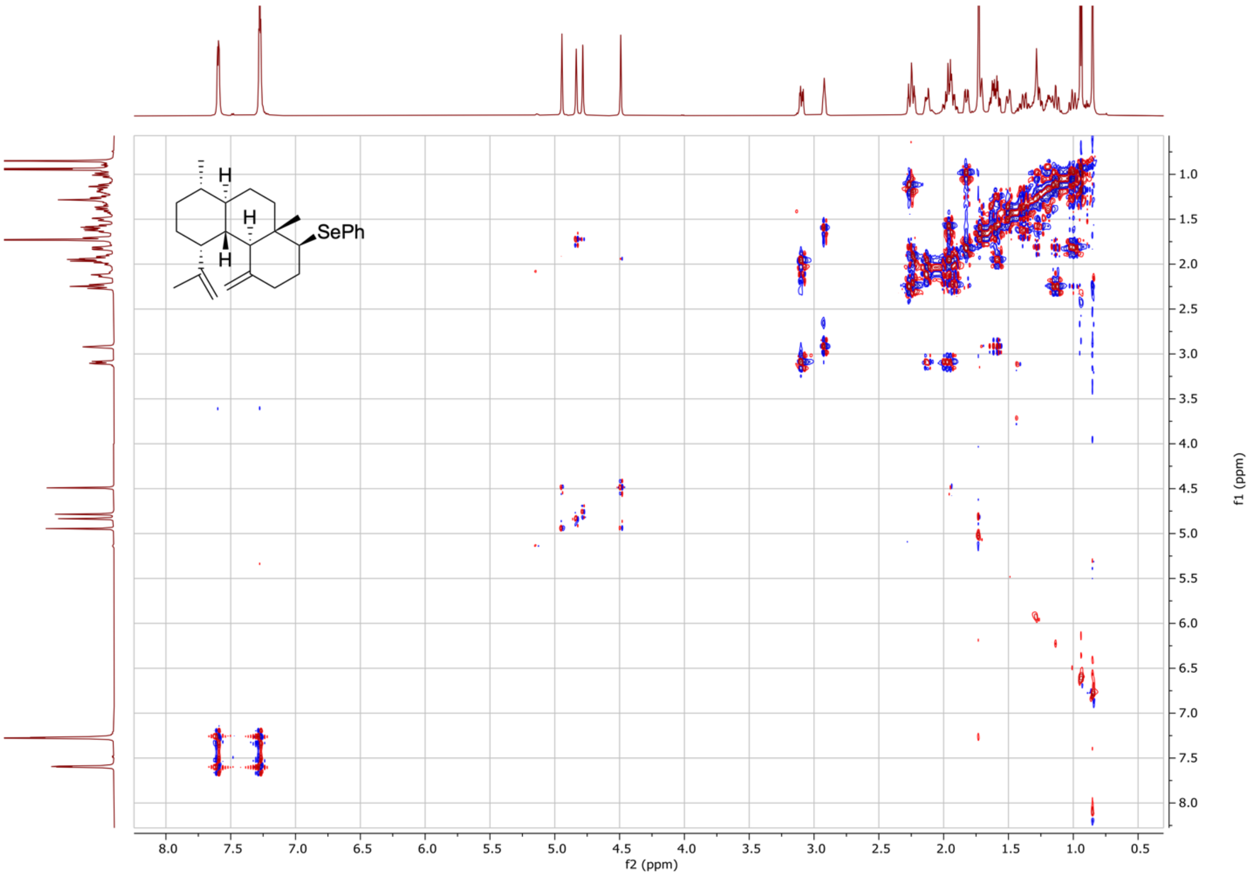

Figure S33. ^{1}H-^{13}C HSQC spectrum of **17** in chloroform-*d* (600 MHz).

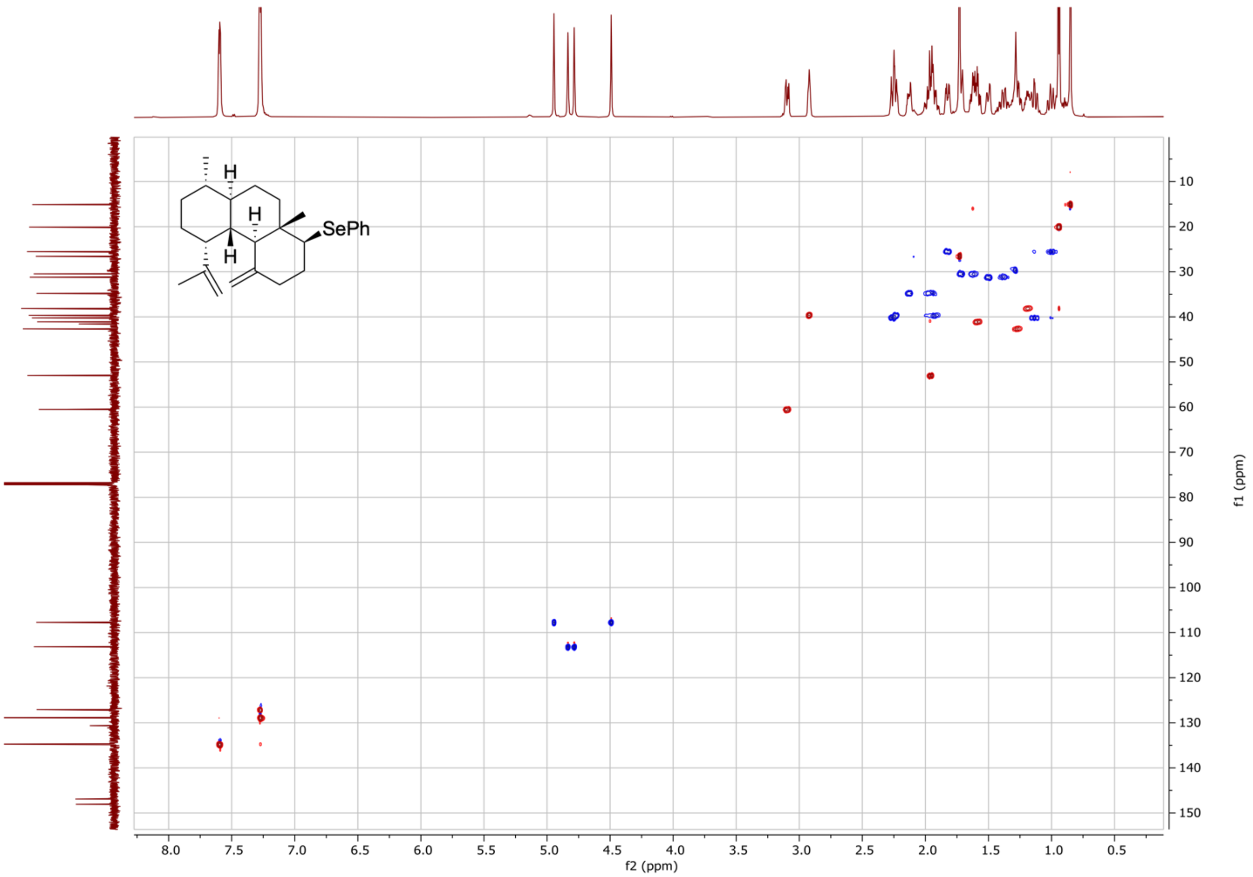

Figure S34. ^{1}H-^{13}C HMBC spectrum of **17** in chloroform-*d* (600 MHz).

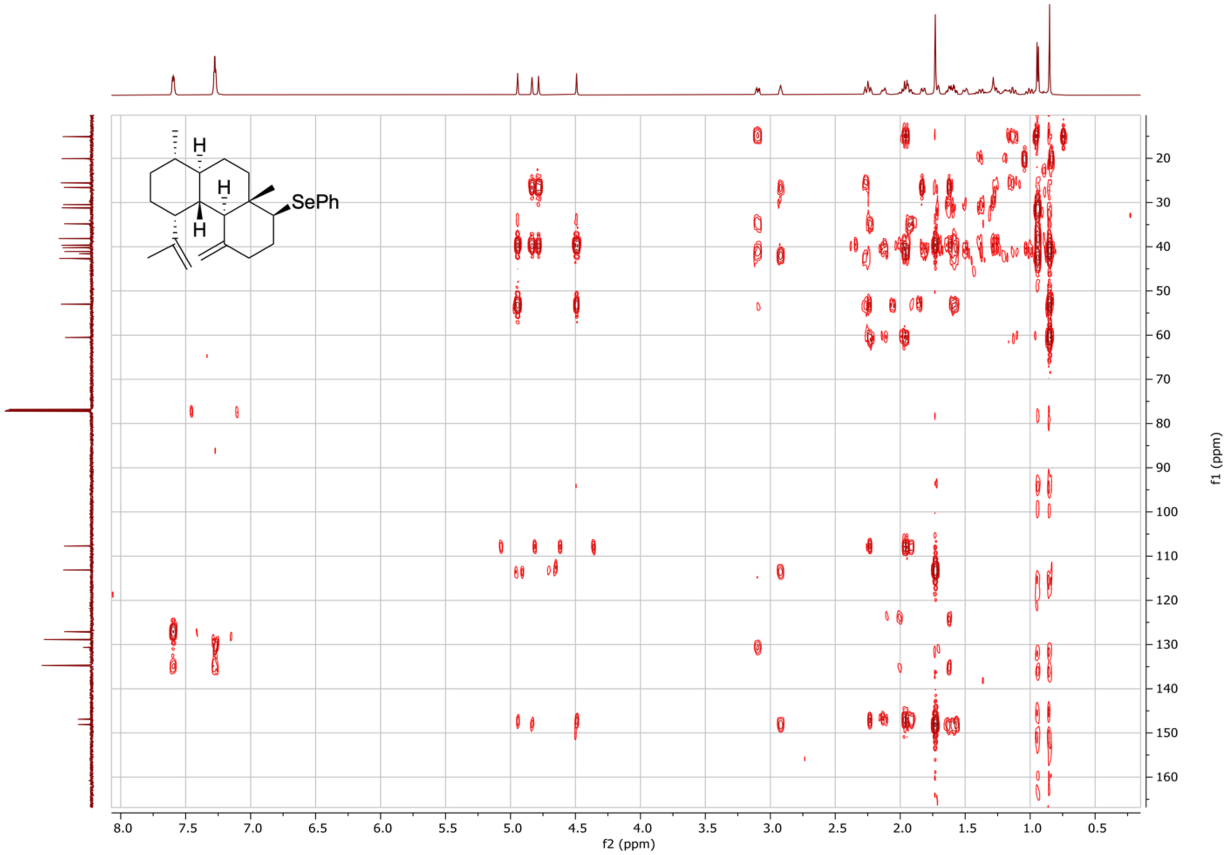

Figure S35. ^{1}H-^{1}H NOESY spectrum of **17** in chloroform-*d* (600 MHz).

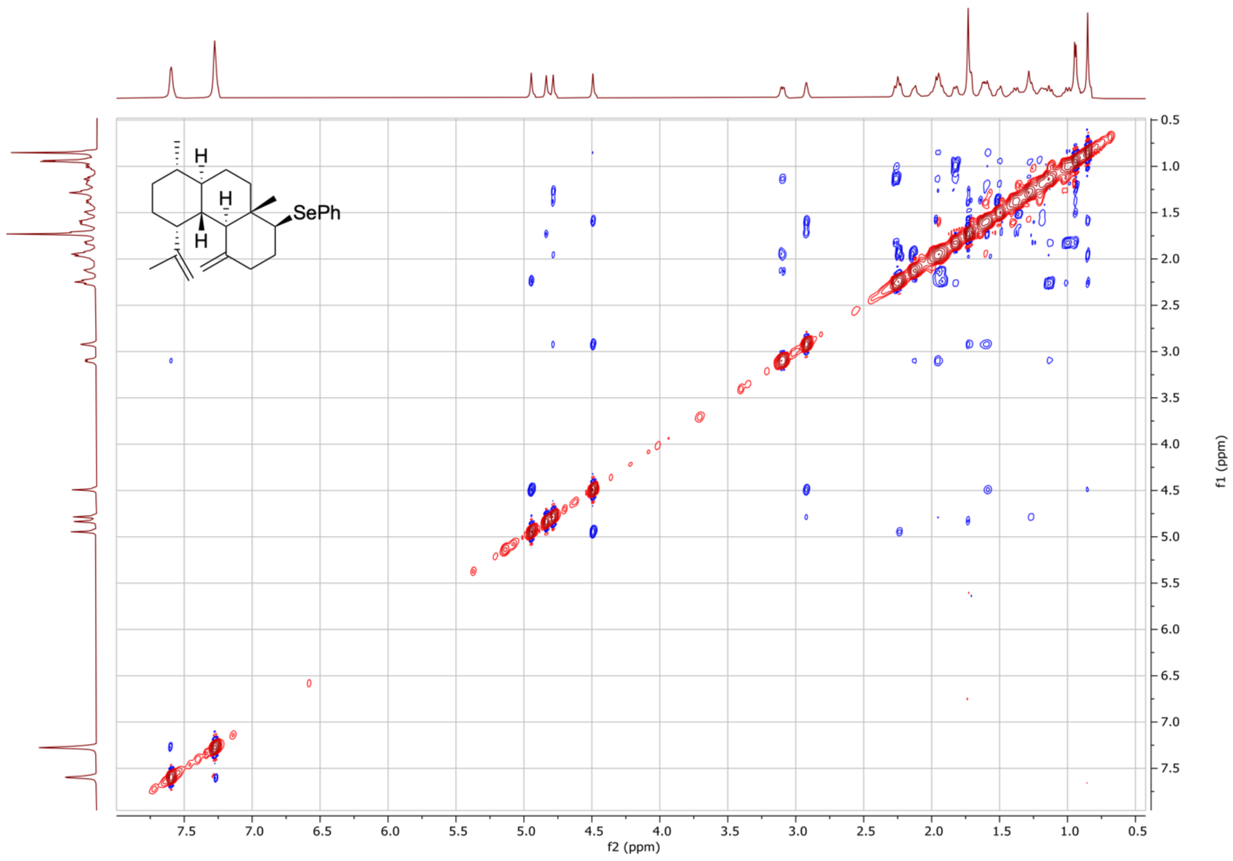

Figure S36. Selective 1D NOESY spectrum of **17** in chloroform-*d* with selective excitation at 3.10 ppm (H-6); mixing time = 300 ms.

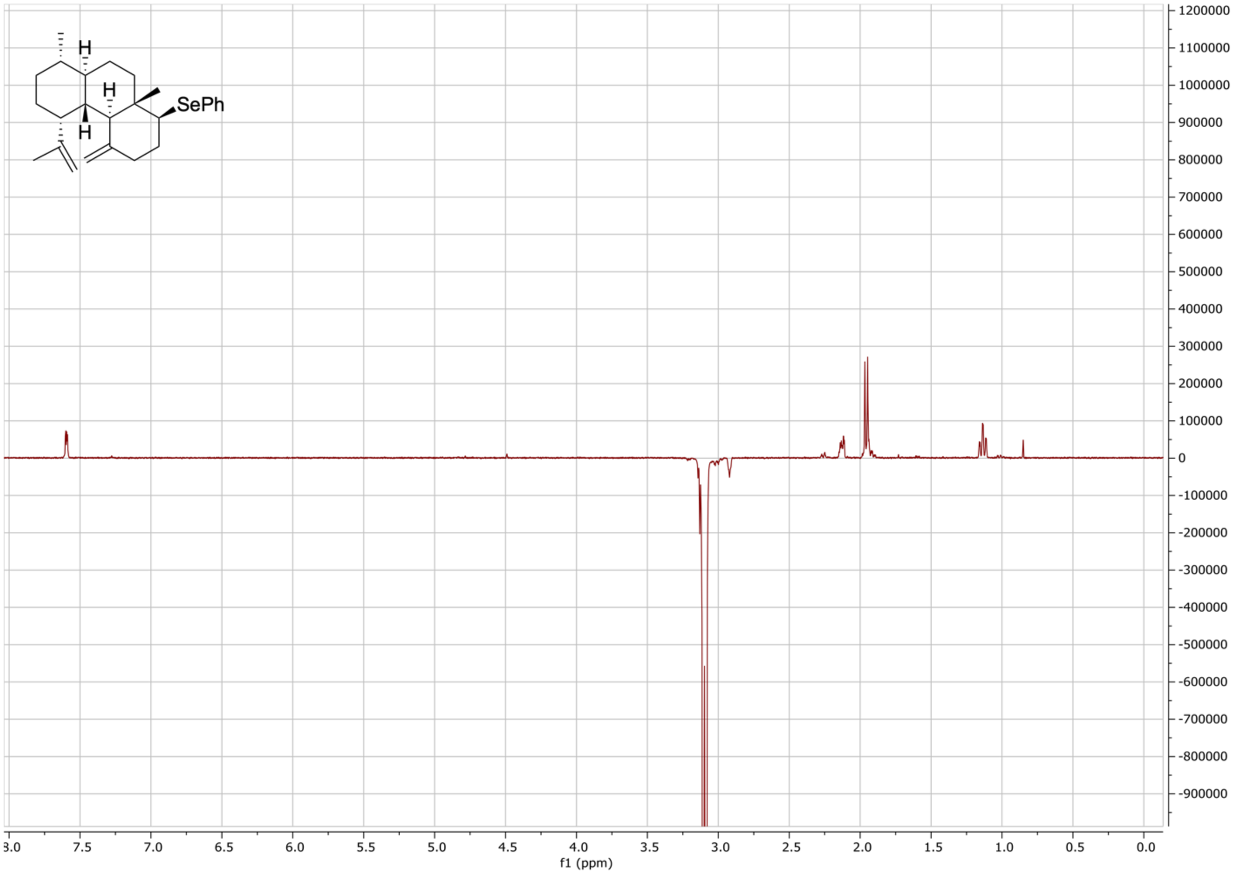

Table S1. Crystal data and structure refinement for **9**

CCDC Deposition Number	2326275
Empirical formula	$C_{20}H_{32}O$
Formula weight	288.45
Temperature/K	100(2)
Wavelength/Å	1.54178
Crystal system	Orthorhombic
Space group	$P2_12_12_1$
a/Å	6.7692(2)
b/Å	10.7877(2)
c/Å	23.7138(5)
α/°	90
β/°	90
γ/°	90
Volume/Å^3	1731.68(7)
Z	4
Density (calculated)/Mg/m^3	1.106
Absorption coefficient/mm^{-1}	0.490
F(000)	640
Crystal size/mm^3	0.192 x 0.130 x 0.091
Θ range for data collection/°	3.728 to 66.633
Index ranges	$-8 \le h \le 8$, $-12 \le k \le 12$, $-28 \le l \le 28$
Reflections collected	20792
Independent reflections	3029 [R_{int} = 0.0250]
Completeness to Θ	99.2%
Absorption correction	Multi-scan
Refinement method	Full-matrix least-squares on F^2
Data / restraints / parameters	3029 / 0 / 194
Goodness-of-fit on F^2	1.062
Final R indices [I>2σ (I)]	R_1 = 0.0268, wR_2 = 0.0673 [2992]
R indices (all data)	R_1 = 0.0272, wR_2 = 0.0678
Absolute structure parameter	-0.01(5)
Largest diff. peak and hole /e Å^3	0.176 and -0.153

Supplementary References

1. Xu, B.; Ning, W.; Wei, X.; Rudolf, J. D. *Org. Biomol. Chem.* **2022**, *20*, 8833–8837. doi:10.1039/D2OB01931K.
2. Li, Z.; Xu, B.; Kojasoy, V.; Ortega, T.; Adpressa, D. A.; Ning, W.; Wei, X.; Liu, J.; Tantillo, D. J.; Loesgen, S.; Rudolf, J. D. *Chem* **2023**, *9*, 698–708. doi:10.1016/j.chempr.2022.12.006.
3. Wang, Z.; Yang, Q.; He, J.; Li, H.; Pan, X.; Li, Z.; Xu, H.-M.; Rudolf, J. D.; Tantillo, D. J.; Dong, L.-B. *Angew. Chem. Intl. Ed.* **2023**, *62*, e202312490. doi:10.1002/anie.202312490.
4. Hu, Y. L.; Zhang, Q.; Liu, S. H.; Sun, J. L.; Yin, F. Z.; Wang, Z. R.; Shi, J.; Jiao, R. H.; Ge, H. M. *Chem. Sci.* **2023**, *14*, 3661–3667. doi:10.1039/D2SC06033G.
5. Sheldrick, G. M. *Acta. Cryst.* **2015**, *C71*, 3–8. https://doi:10.1107/S2053229614024218.
6. Riss, T. L.; Moravec, R. A.; Niles, A. L.; Duellman, S.; Benink, H. A.; Worzella, T. J.; Minor, L. Cell Viability Assays in Assay Guidance Manual, US National Library of Medicine, Bethesda (MD), 2013 (updated 2016).
